# Turning dead leaves into an active multifunctional material as evaporator, photocatalyst, and bioplastic

**DOI:** 10.1038/s41467-023-36783-8

**Published:** 2023-03-02

**Authors:** Siyuan Fang, Xingyi Lyu, Tian Tong, Aniqa Ibnat Lim, Tao Li, Jiming Bao, Yun Hang Hu

**Affiliations:** 1 Department of Materials Science and Engineering, Michigan Technological University, Houghton, MI 49931 USA; 2 Department of Chemistry and Biochemistry, Northern Illinois University, DeKalb, IL 60115 USA; 3 Department of Electrical and Computer Engineering, University of Houston, Houston, TX 77204 USA; 4 X-ray Science Division, Argonne National Laboratory, Lemont, IL 60439 USA

**Keywords:** Materials for energy and catalysis, Green chemistry, Biomaterials, Process chemistry

## Abstract

Large numbers of leaves fall on the earth each autumn. The current treatments of dead leaves mainly involve completely destroying the biocomponents, which causes considerable energy consumption and environmental issues. It remains a challenge to convert waste leaves into useful materials without breaking down their biocomponents. Here, we turn red maple dead leaves into an active three-component multifunctional material by exploiting the role of whewellite biomineral for binding lignin and cellulose. Owing to its intense optical absorption spanning the full solar spectrum and the heterogeneous architecture for effective charge separation, films of this material show high performance in solar water evaporation, photocatalytic hydrogen production, and photocatalytic degradation of antibiotics. Furthermore, it also acts as a bioplastic with high mechanical strength, high-temperature tolerance, and biodegradable features. These findings pave the way for the efficient utilization of waste biomass and innovations of advanced materials.

## Abstract (web summary)

Large numbers of leaves fall off trees each autumn. Here, the authors turn dead leaves into an active material which acts as bioplastic and shows high performance in solar water evaporation, photocatalytic H_2_ production and antibiotic degradation.

## Introduction

**Fig. 1:**
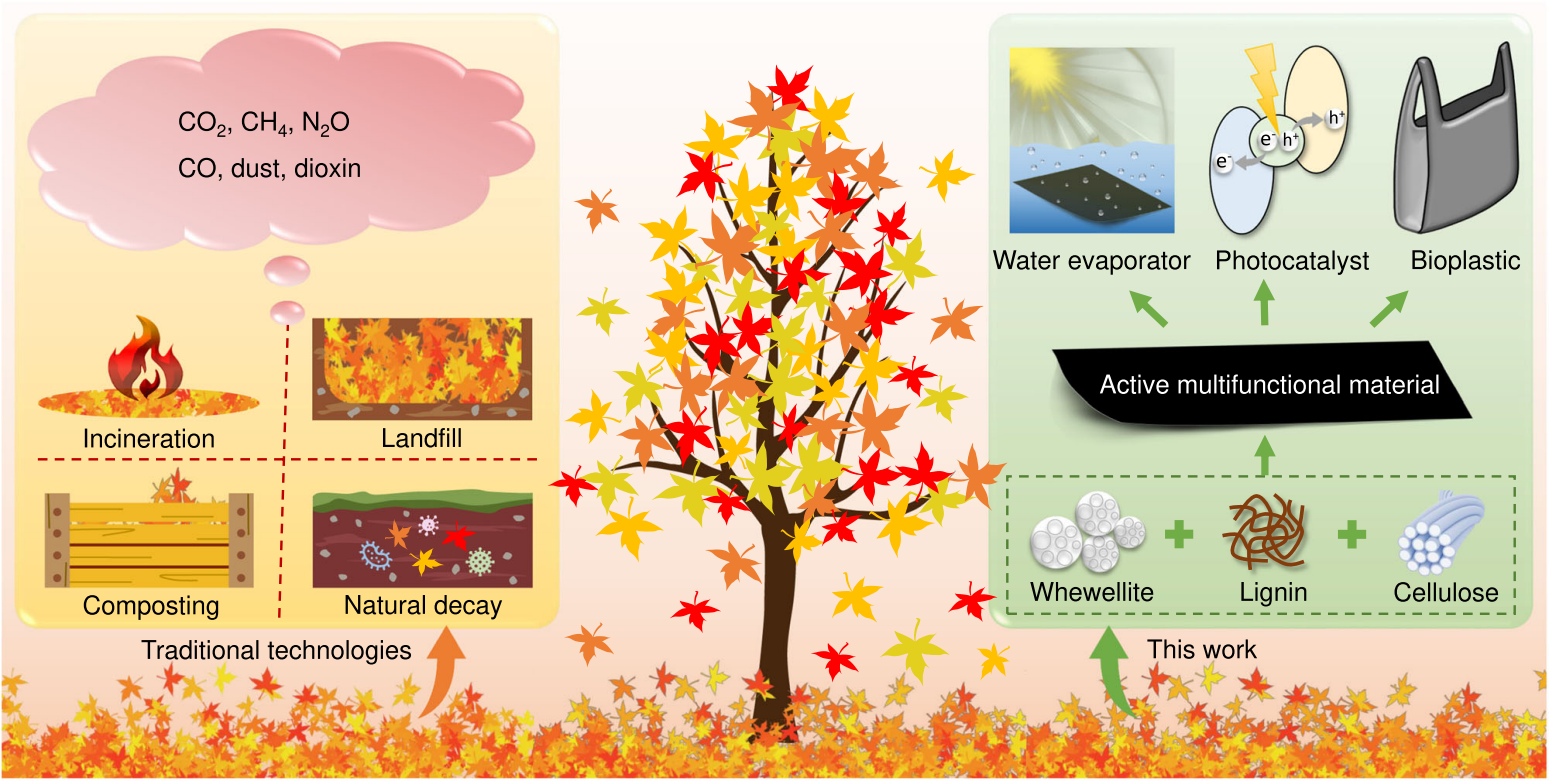
Schematic illustration of technologies for treating dead leaves. Comparison of the approach developed in this work with traditional technologies.

The world has 4.1 billion hectares of tree coverage, which accounts for 31% of the total land area^1^. The global number of trees is approximately 3 trillion, and they make great contributions to carbon sequestration, timber stocks, water and air quality control, etc^2,3^. The leaves constitute one of the most important components of a tree, and they serve as the primary sites of photosynthesis to produce food for the whole tree and play an important role in transpiration via stomata^4^. Given that large numbers of leaves fall off trees over time, it is unfortunate the majority of them ultimately decay naturally into fertilizers, and this process emits greenhouse gases (CO_2_, CH_4_, and N_2_O) into the atmosphere (Fig. 1)^5,6^. In addition to this natural process, waste dead leaves have been mainly treated via incineration, landfilling, and composting; whereas incineration emits large amounts of CO_2_ and noxious gases/particulates, landfilling generates CH_4_ and refractory leachate, and composting requires a long time^7,8^.

In recent years, breakthroughs in the structural reconstruction of wood have been made by Hu and coworkers^9–11^. For example, they demonstrated that wood can be converted into a bioplastic by lignin regeneration^9^ and a moldable lightweight 3D structural material via rapid water shock^10^. In contrast to wood, which is of great value and mainly contains lignocellulose, dead leaves are waste with considerable biominerals that provide great challenges for utilization. Recently, the carbonization of dead leaves was explored as a means to produce carbon materials used as adsorbents for dyes and heavy metals^12,13^ and electrodes for supercapacitors and batteries^14,15^. However, this process consumes high amounts of energy because the biostructures of the leaves are completely destroyed. Therefore, it is urgent to explore strategies for turning waste leaves into useful materials while retaining their biocomponents.

While the organic lignocellulose of leaves has received great attention, calcium oxalate monohydrate (whewellite), a biomineral used for structural support and calcium storage^16–18^, is frequently neglected during biomass utilization. The formation of whewellite sequesters calcium away from the cytoplasm, where the concentration of ionic calcium remains at the micromolar level^18^. As the leaves age, whewellite accumulates and generally constitutes from 1% to 80% of the dry weights of leaves^16,18,19^. This has stimulated us to pay special attention to the whewellite biomineral for turning dead leaves into useful materials.

In this work, we exploited whewellite as a strong binder for lignin and cellulose via extensive chemical bonding and hydrogen bonding, leading to the invention of an active multifunctional material (AMM) from the dead leaves. Furthermore, the optical, thermal, mechanical, and biodegradable properties of the AMM film enable its high performance in solar water evaporation, photocatalytic hydrogen production, photocatalytic degradation of antibiotics and as a high-temperature-resistant bioplastic (Fig. 1). The production of AMM from dead leaves and its wide applications will play important roles in addressing critical energy and environmental issues.

## Results and Discussion

### Synthesis and characterization of AMM

**Fig. 2:**
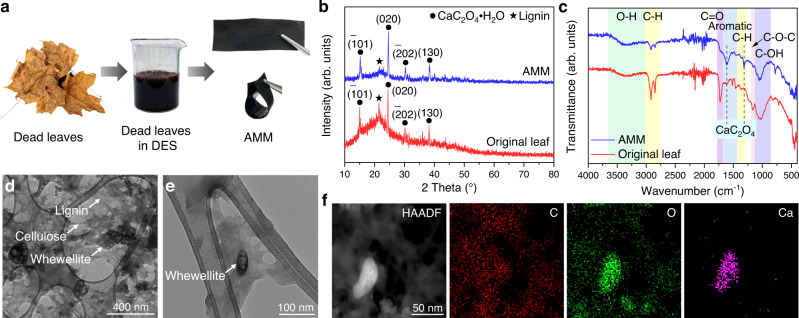
Synthesis and composition analyses of AMM. **a** Visual illustration of the conversion from red maple dead leaves to AMM with deep eutectic solvent (DES). **b** XRD patterns of AMM and original leaf. **c** FT-IR spectra of AMM and original leaf. **d**, **e** TEM images of AMM. **f** EDS elemental maps of AMM.

As illustrated in Fig. 2a, AMM film, which appears black with a high mechanical flexibility and an adjustable thickness (Supplementary Fig. 1), was prepared from the dead leaves of red maple trees with a deep eutectic solvent (DES) composed of choline chloride and oxalic acid dihydrate. Additional information on the synthesis of materials and procedures used in the study are present in the Methods section. In this process, lignin was in-situ regenerated and cellulose was defibrillated^9^. In addition, most of the hemicellulose, pigments, and mineral elements were removed (Supplementary Table 1). The yield of AMM calculated by the dry weight was 57.7%, and lignin, cellulose, and whewellite were the main components. X-ray diffraction (XRD) patterns (Fig. 2b) demonstrated the presence of whewellite in both the AMM and the original leaf, together with lignocellulose, for which broad peaks were located at 10−55°^20^. There were no other crystal impurities (minerals). In addition, the reduced crystallinity of the lignocellulose after DES treatment provided evidence for deconstruction of the dead leaves. Moreover, in the Fourier transform infrared (FT-IR) spectra (Fig. 2c), the signals at 1312 and 1615 cm^−1^ for C = O stretching vibrations of the whewellite were enhanced after the DES treatment^21^, while the signal at 1728 cm^−1^ resulting from C = O stretching vibrations of the acetyl groups and carboxylic acids of hemicellulose was weakened^22^. In addition, the characteristic vibrations for the aromatic skeleton of lignin at 1460 and 1512 cm^−1^, as well as the C-OH and C-O-C stretching vibrations for cellulose at 1030, 1052, and 1157 cm^−1^ were clearly resolved^9,22^. Furthermore, X-ray photoelectron spectra (Supplementary Figs. 2, 3 and Table 2) indicated C, O, and Ca as the main constituent elements of the AMM and increased C = O, O-C-O, and C-OH groups after the DES treatment. These oxygen-containing groups were responsible for the formation of an extensive hydrogen bonding network.

The transmission electron microscopy (TEM) image (Fig. 2d) reveals the coexistence of lignin flakes, cellulose nanofibrils, and whewellite nanoparticles in the AMM. The high-resolution TEM image (Fig. 2e) and energy dispersive spectroscopy (EDS) elemental maps (Fig. 2f) further demonstrated that whewellite nanoparticles were embedded in lignocellulose with intimate contacts. To provide more insights into the molecular interactions of the AMM, density functional theory (DFT) calculations were conducted for the lignin-cellulose-whewellite composite (Supplementary Fig. 4 and Supplementary Data 1). The binding energy reached 468.9 kJ mol^−1^, which was 25 times the binding energy of the lignin-cellulose composite (18.9 kJ mol^−1^, Supplementary Fig. 5 and Supplementary Data 2). This suggested the key role of whewellite as the binder for lignin and cellulose, which distinguished the leaves from other biomasses. Moreover, the electrostatic potential surfaces of the constituents overlapped substantially with each other and showed complementary features, implying strong intermolecular interactions and the formation of local electrostatic fields that can drive charge migration right after excitation^23^. The close-up views (Supplementary Fig. 4b, c) revealed that -OH groups in lignin and cellulose formed hydrogen bonds with O atoms in oxalate. Furthermore, the electron density difference plots (Supplementary Fig. 4d, e) indicated the formation of chemical bonds (bond length: ~2.5 Å) between the Ca atoms and -OH groups in lignin and cellulose with directional electron redistribution.

**Fig. 3:**
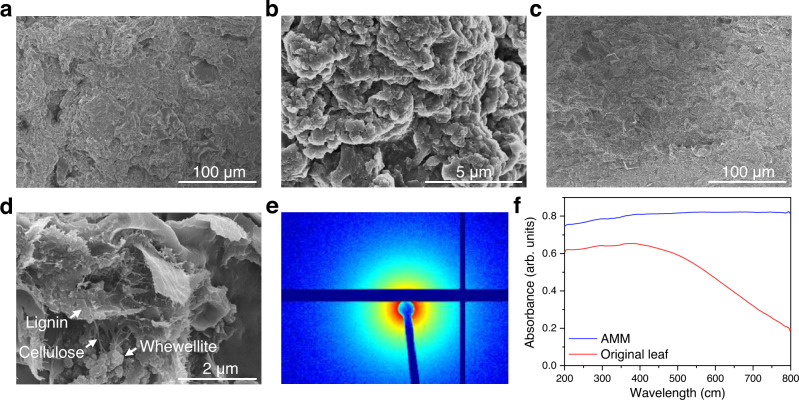
Morphology, structure, and light absorption of the AMM film. **a**, **b** Top-view SEM images of the AMM film. **c**, **d** Cross-sectional SEM images of the AMM film (after tension test). **e** SAXS pattern for the AMM film. **f** UV‒vis spectra of the AMM film and original leaf.

The morphological structure of the AMM film was evaluated by scanning electron microscopy (SEM). The top-view SEM images (Fig. 3a, b) suggested a rough surface consisting of irregular particles, while the cross-sectional SEM images (Fig. 3c, d) demonstrated a denser structure with a number of cellulose nanofibrils across the lignin flakes and whewellite nanoparticles. The density of the AMM film was calculated as 1021 kg m^−3^, twice that of the original leaf (516 kg m^−3^), which possessed a loose structure and a smooth surface (Supplementary Fig. 6). Moreover, as illustrated in Fig. 3e, the small angle X-ray scattering (SAXS) pattern verified the isotropic structure of the AMM film without specific alignments. The rough surface of the AMM film allowed efficient internal reflection of photons, which greatly enhanced the absorption of incident light^24^. Ultraviolet‒visible (UV‒vis) (Fig. 3f) and Fourier transform near-infrared (FT-NIR) (Supplementary Fig. 7) spectra exactly confirmed the strong absorption of light from UV across visible to NIR regions, in great contrast to the original leaf that only absorbed UV and some of the visible light. This broad light absorption by the AMM is due to extensive conjugation of the lignocellulose since the removal of the minerals and pigments demonstrated negligible effects on the light absorption (Supplementary Fig. 8). This implied the great promise of AMM for use in photothermal and photocatalytic applications.

### AMM as the evaporator, photocatalyst, and bioplastic

**Fig. 4:**
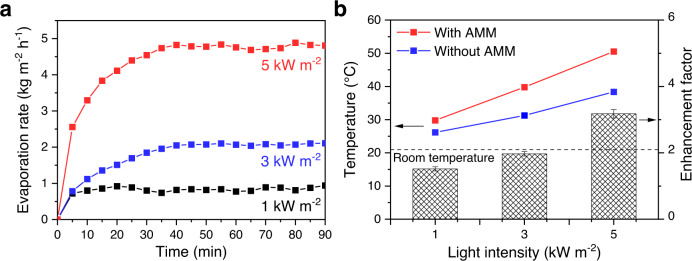
Application of the AMM film for solar water evaporation. **a** Solar water evaporation rate over the AMM film under simulated sunlight at various light intensities. **b** Water temperature under simulated sunlight and enhancement factor for the solar water evaporation rate with regards to pure water.

Solar water evaporation is an attractive technology for water purification, steam sterilization, and electricity generation due to its high energy efficiency and low environmental impact^25,26^. As a promising photothermal material, the AMM film was used for solar water evaporation, and it realized a high evaporation rate of 0.8 kg m^−2^ h^−1^ (Fig. 4a) and an impressive solar-to-steam efficiency of 52.5% at ambient solar light intensity (1 kW m^−2^), which are among the highest values reported for state-of-the-art materials^26–28^. This mainly resulted from the lignocellulose instead of the minerals and pigments (Supplementary Table 3), consistent with the light absorption characteristics discussed above. Further amplifying the light intensity to 3 and 5 kW m^−2^ increased the rates of evaporation over the AMM film to 2.1 and 4.8 kg m^−2^ h^−1^, respectively (Fig. 4a). Moreover, as shown in Fig. 4b, the enhancement factor relative to the blank test without AMM reached 1.5, 2.0, and 3.2 at 1, 3, and 5 kW m^−2^, respectively, owing to the elevated temperature by the AMM, which was significant, especially at high light intensities.

**Fig. 5:**
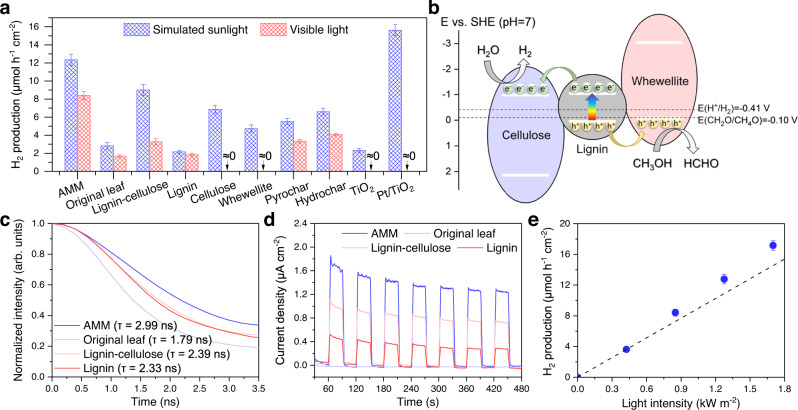
Application of the AMM film for photocatalytic hydrogen production and analysis of charge carrier behaviors. **a** Photocatalytic hydrogen production from a methanol-water mixture under simulated sunlight (1 kW m^−2^) or visible light (λ > 400 nm, 0.85 kW m^−2^). **b** Schematic band structure of the AMM and visible-light photocatalytic hydrogen production over it. **c** Time-resolved fluorescence decay curves. **d** Transient photocurrent response under visible light (alternating light-on for 30 s and light-off for 30 s). **e** Photocatalytic hydrogen production from a methanol-water mixture over the AMM film under visible light at various intensities.

The photothermal effect is mainly caused by indirect nonradiative relaxation of electron-hole pairs, but if some photogenerated electrons and holes can be quickly captured by reactant molecules before recombination, photocatalytic reactions occur^29^. Here, we conducted the photocatalytic hydrogen production reaction with a methanol-water mixture over the AMM film to demonstrate its catalytic capability for clean fuel generation. The use of methanol enabled favorable thermodynamics. As shown in Fig. 5a, the hydrogen production rate over the AMM film under simulated sunlight irradiation (1 kW m^−2^) was as high as 12.4 μmol h^−1^ cm^−2^, which was 4.2 times that over the original leaf. After filtering the UV light, the hydrogen production rate still reached 8.4 μmol h^−1^ cm^−2^, marking a noble-metal-free and visible-light-responsive photocatalyst that outperformed dead leaves-derived pyrochar and hydrochar and realized an efficiency on the same order of magnitude as that of the prototypical water splitting photocatalyst, 1 wt% Pt/TiO_2_ (15.6 μmol h^−1^ cm^−2^ and 0 under simulated sunlight and visible light, respectively). Compared to the AMM, the constituents lignin, cellulose, and whewellite, as well as the lignin-cellulose composite exhibited poorer photocatalytic efficiencies under simulated sunlight and visible light irradiation, implying that these three components might form heterostructures that allow effective separation of photogenerated electrons and holes. The trace pigments in the AMM made little contribution, as evidenced by the similar activity seen without them (Supplementary Table 4).

To demonstrate the above hypothesis, the band structures of lignin, cellulose, and whewellite were determined (see Supplementary Figs. 9-12 and Table 5). As shown in Fig. 5b, cellulose possesses the lowest conduction band and whewellite has the highest valence band, which are energetically favorable for the accumulation of electrons and holes, respectively. Moreover, due to the large band gaps of cellulose and whewellite (3.12 and 2.96 eV), they can only be excited by UV light, while the small band gap of lignin (1.08 eV) allows its absorption of visible light. Under visible light irradiation, electrons from the top of the lignin valence band are excited to the bottom of its conduction band, leaving holes in the valance band. The generated electrons and holes would be transferred to the cellulose and whewellite, respectively, realizing effective separation. This was confirmed by the measurements of time-resolved fluorescence decay (Fig. 5c) and transient photocurrent (Fig. 5d). Namely, both the fluorescence lifetimes and the transient photocurrent intensities increased in the order original leaf <lignin <lignin-cellulose composite <AMM, indicating the vital importance of the lignin, cellulose, and whewellite constituents in the AMM and their strong interactions. The long radiative lifetime (slow decay kinetics) of charge carriers in the AMM enhanced their probabilities of participating in photocatalytic reactions before recombination, and the large photocurrent intensity of the AMM provided direct evidence for the ample availability of active charge carriers^29^.

After charge carrier separation and migration, the reduction of water and the oxidation of methanol, whose redox potentials exactly meet the thermodynamic requirements, occurred on the surfaces of the cellulose and whewellite, respectively. Despite the favorable thermodynamics, the oxidation driving force (energy difference between the oxidation potential of methanol and the top of the valence band for whewellite) is as small as 0.02 V, implying poor kinetics for this reaction^24,30,31^. However, the temperature rise induced by the photothermal effect (Supplementary Fig. 13a) helped by activating the reactant molecules^30,31^. As shown in Fig. 5e, the hydrogen production rate demonstrated a superlinear increase with the light intensity, which might have resulted from either a thermal catalytic reaction or acceleration of the photocatalytic reaction by thermal energy. Therefore, we conducted dark control tests at elevated temperatures up to 70 °C but did not detect any hydrogen (Supplementary Fig. 13b). This confirmed the contribution of the photothermal effect to the photocatalytic performance of water splitting, though alternative pathways such as methanol reforming cannot be ruled out. Furthermore, the hydrogen production efficiency remained unchanged after 5 test cycles (Supplementary Fig. 14), suggesting the stability and recyclability of the AMM film.

**Fig. 6:**
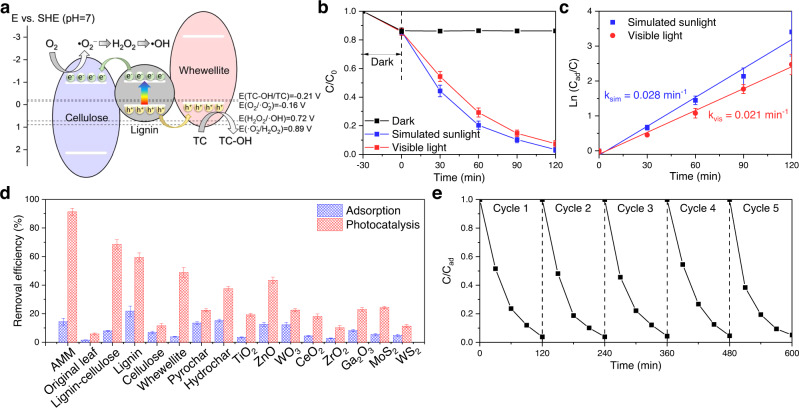
Application of the AMM film for photocatalytic degradation of the antibiotic tetracycline. **a** Schematic for the visible-light photocatalytic degradation of tetracycline (TC) over the AMM film. **b** Removal of tetracycline in the dark or under simulated sunlight (1 kW m^−2^) or visible light (λ > 400 nm, 0.85 kW m^−2^) over the AMM film. **c** Reaction kinetic analyses for the photocatalytic degradation of tetracycline over the AMM film. **d** Comparison of the tetracycline removal efficiencies via adsorption and visible-light photocatalysis with those for a variety of other materials. **e** Cycling performance of the photocatalytic tetracycline degradation process over the AMM film under simulated sunlight.

In addition to hydrogen production, degradation of aqueous organic pollutants such as dyes and antibiotics constitutes another important application for photocatalysis^32–34^. Since photocatalytic degradation mainly relies on reactive oxygen species, a favorable band alignment is essential. Fortunately, as shown in Fig. 6a, the more negative conduction band of the cellulose allows sequential reductions of O_2_ to generate •O_2_^−^, H_2_O_2_, and •OH. Furthermore, the photogenerated holes can directly oxidize the pollutants (here, the antibiotic tetracycline was used as an example). Consequently, 96.7% and 92.6% of the tetracycline was removed by the AMM film during 2 h photocatalytic reactions run under simulated sunlight and visible light, respectively (Fig. 6b). Furthermore, the kinetic analysis revealed the first-order reactions with large rate constants of 0.028 min^−1^ and 0.021 min^−1^ under simulated sunlight and visible light, respectively (Fig. 6c). Compared to its constituents, the original leaf, pyrochar, hydrochar, and other widely applied inorganic semiconductors, the AMM film exhibited ultrahigh efficiencies for both adsorption and visible-light photocatalysis (Fig. 6d), demonstrating the superiority of this three-component heterostructure.

Furthermore, the pathway for photocatalytic tetracycline degradation was provided in Supplementary Fig. 15 based on liquid chromatography‒mass spectrometry analysis, suggesting that hydroxylated tetracycline was the primary product generated via hole oxidation and it was sequentially converted into small molecules. The toxicities of these small molecules (P5 and P6 in Supplementary Fig. 15) are significantly lower than that of tetracycline (Supplementary Fig. 16). More importantly, the AMM film could be reused after simply flushing the surface with deionized water, and it repeatedly showed high degradation efficiencies above 95% (Fig. 6e). Together, these results demonstrated the great promise of the AMM film for environmental remediation.

**Fig. 7:**
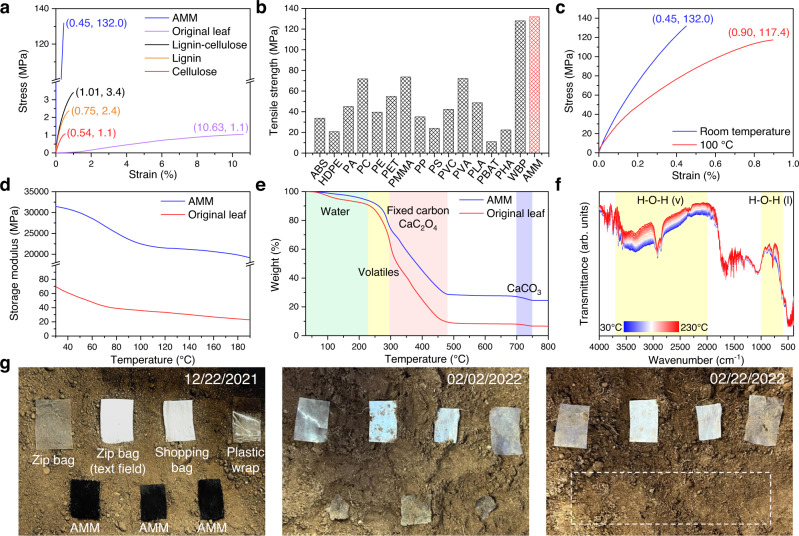
Application of the AMM film as a bioplastic and its mechanical, thermal, and biodegradation properties. **a** Stress‒strain curve of the AMM in comparison with those for its components and the original leaf. **b** Comparison of the tensile strengths of AMM, wood-derived bioplastic (WBP), and petroleum-based plastics including acrylonitrile butadiene styrene (ABS), high-density polyethylene (HDPE), polyamide (PA), polycarbonate (PC), polyethylene (PE), polyethylene terephthalate (PET), polymethyl methacrylate (PMMA), polypropylene (PP), polystyrene (PS), polyvinyl chloride (PVC), polyvinyl alcohol (PVA), polylactide (PLA), polybutylene adipate co-terephthalate (PBAT), and polyhydroxyalkanoate (PHA). **c** Stress‒strain curve of the AMM at 100 °C in comparison with that at room temperature. **d** Temperature-dependent storage modulus of AMM and original leaf. **e** TG curves of AMM and original leaf in air. **f** In-situ DRIFTS spectra of the AMM in air. **g** Biodegradability tests of the AMM and frequently used polyethylene plastics in soil.

Beyond these applications in energy and environment, the AMM also acted as a high temperature-resistant bioplastic. This was supported by its mechanical properties, thermal stability, and biodegradability. The tensile strength of the AMM reached 132 MPa, which was two orders of magnitude higher than those of the original leaf, lignin-cellulose composite, lignin, and cellulose films (Fig. 7a) and much higher than those of petroleum-based plastics (Fig. 7b and Supplementary Table 6). Additionally, the Young’s modulus and toughness were 50402 MPa and 344 kJ m^−3^, respectively, far surpassing those of other materials (Supplementary Table 7). The substantial mechanical robustness of the AMM film was due to binding of the lignocellulose by the whewellite, as suggested by the cross-sectional SEM image of the fractured AMM (Fig. 3d) and the DFT calculations discussed above. Moreover, at 100 °C, the tensile strength, Young’s modulus, and toughness of the AMM could still reach 117 MPa, 39004 MPa, and 686 kJ m^−3^, respectively, with twice the elongation at break (Fig. 7c), indicating its enormous potential for high-temperature applications.

Moreover, dynamic mechanical analyses were performed on the AMM film and the original leaf. As shown in Fig. 7d, the room-temperature storage modulus of the AMM film reached 31448 MPa, which was 450 times that of the original leaf (70 MPa), implying significantly enhanced rigidity and load-bearing capability^35^. Due to the softening effect of the polymeric materials (lignocellulose)^36^, the storage modulus was reduced with increasing temperature from 30 to 190 °C, while the reduction over the AMM film was just 39.0%; this was much smaller than the 67.1% reduction seen for the original leaf, revealing the improved thermomechanical properties of the AMM. Moreover, the glass transition temperature of the AMM was estimated at 105 °C based on the peak for the temperature-dependent damping factors (Supplementary Fig. 17).

The thermal stability of the AMM was investigated via thermogravimetric (TG) analysis and in-situ diffuse reflectance infrared Fourier transform spectroscopy (DRIFTS). The TG curves (Fig. 7e) showed that AMM and the original leaf exhibited similar decomposition temperature windows ranging from 230 to 480 °C for the volatiles, fixed carbon, and calcium oxalate, while the AMM demonstrated lower decomposition rates (Supplementary Fig. 18). Moreover, the amount of solid residue remaining after pyrolysis of the AMM (24.4 wt%) was much higher than that of the original leaf (6.6 wt%), indicating the highly crosslinked nature of the AMM^37,38^. In-situ DRIFTS spectra (Fig. 7f) further evidenced the stability of the AMM up to 230 °C without attenuation of the characteristic signals except for the two broad bands corresponding to water stretching and libration vibrations^21^. Further elevation of the temperature to 600 °C destroyed the C-H bonds of the lignocellulose and converted the calcium oxalate to calcium carbonate (Supplementary Fig. 19), consistent with the TG results. Together, these results proved the thermal stability of the AMM below 230 °C.

Furthermore, the biodegradabilities of the AMM and daily used polyethylene plastics (zip bag, shopping bag, and plastic wrap) were compared by burying them in soil at a depth of 10 cm. The AMM became brittle and fractured after 40 days and completely disappeared after 2 months (Fig. 7g). Decomposition of the AMM might have occurred via three interacting processes, namely, fragmentation by soil fauna, mineralization and humification of the lignocellulose and whewellite mediated by microorganisms, and leaching of the soluble compounds into the soil^16,39,40^. In contrast, all of the daily used polyethylene plastics remained unchanged after 2 months, which poses a severe threat to the environment. The life cycle assessment (Supplementary Table 8) further demonstrated the smaller environmental impact of the AMM compared to those of the petroleum-based plastics and wood-derived bioplastic^9^.

### Universality and advantages of turning dead leaves into AMM

The present approach to turning dead leaves into AMM films exhibited strong universality since various dead leaves, such as those of the *Cercis canadensis*, *Quercus rubra*, and *Acer platanoides*, can be used as raw materials (Supplementary Fig. 20) despite the different proportions of their components^41^. The AMM films prepared from various dead leaves had similar mechanical strengths and demonstrated similar efficiencies for solar water evaporation, photocatalytic hydrogen production, and photocatalytic tetracycline degradation (Supplementary Table 9). In addition, using the separated leaf pulp and leaf veins of the red maple as raw materials led to the formation of AMM films with similar properties and capabilities (Supplementary Table 10), suggesting the great flexibility of the leaf architectures and eliminating the need for disassembling the dead leaves. More importantly, as a trash-to-treasure practice, this approach shows great advantages in carbon emissions relative to the traditional incineration, landfilling, and composting technologies used to dispose of dead leaves (Supplementary Table 11).

In summary, through structural reconstruction and utilization of the whewellite biomineral as a strong binder for lignocellulose, we turned waste leaves into a three-component AMM film. Owing to the intense optical absorption from UV across visible to NIR regions of the solar spectrum and the heterogeneous architecture that enables effective charge separation, the AMM film demonstrated advantages in solar water evaporation, photocatalytic hydrogen production, and photocatalytic antibiotic degradation. Furthermore, due to its desirable mechanical, thermal, and biodegradable properties, the AMM is a promising substitute for petroleum-based plastics and can even withstand high temperatures up to 230 °C. This method for turning waste into wealth provides opportunities for material innovations and sustainable development.

## Methods

### Materials

Dead leaves of red maple (*Acer rubrum*) were collected from the ground in Houghton, Michigan, in October 2021. The leaves were thoroughly washed with pure water, laid out to dry at room temperature, and then ground into powders (≤300 μm). Three other types of dead leaves, namely, those of the *Cercis canadensis*, *Quercus rubra*, and *Acer platanoides*, were collected and treated in the same manner. Choline chloride (≥98%), oxalic acid dihydrate (98%), calcium oxalate monohydrate (99%), hydrochloric acid (36% solution), nitric acid (68-70% solution), and hydrogen peroxide (30% solution) were purchased from Thermo Fisher Scientific. Sodium sulfate (≥99%), sodium sulfite (≥98%), sodium hydroxide (≥97%), sodium chlorite (80%), sulfuric acid (95-98%), acetic acid (≥99.7%), tetracycline (98.0-102.0%), methanol (≥ 99.9%), chloroform (≥99.8%), magnesium oxide (97%), chloroplatinic acid hexahydrate (≥37.50% Pt basis), zinc oxide (99.99%), titanium (IV) oxide (P25, ≥99.5%), tungsten (VI) oxide (<100 nm particle size), cerium (IV) oxide (99.95%), zirconium (IV) oxide (99%), gallium (III) oxide (≥99.99%), molybdenum (IV) sulfide (99%), and tungsten (IV) sulfide (99%) were acquired from Sigma Aldrich.

### Synthesis of the AMM

69.8 g of choline chloride and 63.0 g of oxalic acid dihydrate (at a molar ratio of 1:1) were mixed and melted at 80 °C under stirring to form a transparent deep eutectic solvent. Red maple leaf powder (8.8 g) was added to the solvent and heated to 100 °C for 30 min, after which 13 mL of deionized water was introduced, and the mixture was held at 100 °C for another 2 h. Continuous magnetic stirring was employed during this process. After cooling to room temperature, the resulting solid material was collected by centrifugation, repeatedly washed with deionized water, and then dispersed in 150 mL of deionized water. The dispersion was ultrasonicated at 500 W for 30 min and subsequently centrifuged to give a black slurry. The black slurry was spread on a stainless steel plate to acquire the AMM after water evaporation at room temperature. The yield of AMM calculated by the dry weight was 57.7%. The dead leaves of *Cercis canadensis*, *Quercus rubra*, and *Acer platanoides* trees, as well as the leaf pulp and leaf veins of the red maple tree (separated by hand), were also used as raw materials with which to synthesize the AMM via the same method.

### Preparation of the control samples

The AMM without whewellite or other minerals (named lignin-cellulose) was obtained with a pretreatment procedure used to remove the metal salts from the leaf powders. Namely, 1 g of leaf powder was dispersed in 100 mL of 2 M hydrochloric acid and heated at 70 °C for 3.5 h^42^. The resulting solid was thoroughly washed with deionized water until the pH reached 7 and then dried at 60 °C overnight. This solid, a substitute for the original leaf powders, was utilized to prepare the lignin-cellulose sample by following the same synthetic route described in the last section.

The lignin sample was acquired by using sulfuric acid to treat the leaf powders^43^. Namely, 1 g of leaf powder and 20 mL of a 72 wt% sulfuric acid solution were mixed and stirred at room temperature for 2 h. Afterward, 750 mL of deionized water was introduced and boiled at 100 °C for 4 h. Finally, the solid was washed and dried at room temperature.

The cellulose sample was prepared via two steps^44^. First, 1 g of the leaf powder was added to a 10 mL aqueous solution containing 0.4 M sodium sulfite and 2.5 M sodium hydroxide, which was then heated at 100 °C for 3 h. After repeated washing to remove the residual chemicals, the solid was dispersed in 10 mL of 30% hydrogen peroxide solution and boiled at 100 °C for 3 h until the color of the solid completely disappeared. The solid was then washed and dried at room temperature. The structures of these control samples were confirmed by their XRD patterns (Supplementary Fig. 21) and FT-IR spectra (Supplementary Fig. 22).

In addition, two kinds of biochars, namely, hydrochar and pyrochar, were prepared for comparison with the AMM. The preparation methods were the same as those employed in a previous publication^45^. Namely, the hydrochar was synthesized by stirring a suspension consisting of 7 g of leaf powder and 50 mL of deionized water for 2 h at room temperature, followed by hydrothermal treatment in a Teflon-lined stainless steel autoclave at 200 °C for 5 h. The resulting solid was washed with deionized water and dried at 60 °C overnight. In contrast, the pyrochar was prepared by pyrolyzing the leaf powders at 450 °C for 3 h under a 10 mL min^−1^ argon gas flow.

### Determination of the chemical compositions

The amounts of cellulose, hemicellulose, and lignin in the AMM and original leaf were determined via the approaches described in the literature^9,43,46^. In brief, 1 g of dried AMM or the original leaf powders, 5 mL of 5 wt% sodium chlorite solution, and 2 mL of acetic acid were mixed and heated at 90 °C for 5 h. The resulting solid was thoroughly washed with deionized water, dried at 60 °C overnight, and then weighed to determine the mass of holocellulose (m_1_). Furthermore, the holocellulose solid was dispersed in 20 mL of 10 wt% sodium hydroxide solution and heated at 75 °C for 5 h, followed by washing and drying at 60 °C. The mass of the dried solid (m_2_) represented the cellulose content, while the difference between these two masses (m_1_ − m_2_) indicated the hemicellulose content. The lignin content was determined by using the same methodology described for lignin sample preparation (in the last section), and the dry weight of the final product (m_3_) was utilized for the calculations. Three parallel experiments were carried out for each sample.

1 $${{{{{\rm{Mass}}}}}}\; {{{{{\rm{content}}}}}}\; {{{{{\rm{of}}}}}}\; {{{{{\rm{cellulose}}}}}}=\frac{{{{{{{\rm{m}}}}}}}_{2}{{{{{\rm{g}}}}}}}{1{{{{{\rm{g}}}}}}}\times 100\%$$

2 $${{{{{\rm{Mass}}}}}}\; {{{{{\rm{content}}}}}}\; {{{{{\rm{of}}}}}}\; {{{{{\rm{hemicellulose}}}}}}=\frac{({{{{{{\rm{m}}}}}}}_{1}-{{{{{{\rm{m}}}}}}}_{2}){{{{{\rm{g}}}}}}}{1{{{{{\rm{g}}}}}}}\times 100\%$$

3 $${{{{{\rm{Mass}}}}}}\; {{{{{\rm{content}}}}}}\; {{{{{\rm{of}}}}}}\; {{{{{\rm{lignin}}}}}}=\frac{{{{{{{\rm{m}}}}}}}_{3}{{{{{\rm{g}}}}}}}{1{{{{{\rm{g}}}}}}}\times 100\%$$

The pigments, including chlorophylls, carotenoids, flavonoids, and anthocyanins, were quantified spectrophotometrically^47,48^. Namely, 100 mg of the dried AMM or original leaf powder was mixed with 100 mg of magnesium oxide, followed by grinding in a chloroform-methanol liquid mixture (at a volume ratio of 2:1) for 1 h. Afterward, the homogenate was filtered with a 0.22 μm filtration membrane to obtain a clear extract, to which deionized water (1/5 of the total extract volume) was added. The liquid mixture was well blended by stirring for 30 min and then centrifuged at 2000 g for 10 min until the phases separated. The concentrations of the chlorophylls and carotenoids in the lower chloroform phase were determined with a Shimadzu UV-2450 UV‒visible spectrometer using the equations reported by Wellburn^49^. The flavonoids in the upper aqueous methanol phase were quantified spectrophotometrically by using the absorption coefficient ε_358 nm_ = 25.4 mM^−1^ cm^−1^. Afterward, the aqueous methanol phase was acidified with hydrochloric acid to reach a final acid concentration of 0.1 vol% and then used for quantifying the anthocyanins by measuring the absorbance with the absorption coefficient ε_530 nm_ = 30 mM^−1^ cm^−1^. The solid remaining on the filtration membrane was collected, reacted with hydrochloric acid (to remove the magnesium oxide), washed with deionized water, dried at 60 °C overnight, and used as the raw material for the DES treatment to obtain the sample named “AMM without pigments”.

The mineral elements (K, Ca, Na, Mg, Fe, and Mn) in the AMM and the original leaf were analyzed with a Perkin Elmer Optima 7000DV inductively coupled plasma-optical emission spectrometer (ICP‒OES). The samples were prepared by incineration and acid-dissolution^50^. Namely, 200 mg of the dried AMM or original leaf powders were charred in a muffle furnace at 500 °C for 2 h. After cooling, 0.5 mL of deionized water and 4 mL of nitric acid were added, and the suspension was stirred on a hot plate at 100 °C until dry. Then, the solid was calcined at 500 °C for 1 h, and the resulting ash was dissolved in 10 mL of 20 vol% hydrochloric acid. The samples were diluted 5 times for measurements after 3 days of complete dissolution.

### Characterizations

The crystal structures of the samples were determined with a Scintag XDS2000 X-ray powder diffractometer. The morphological structures of the samples were revealed with an FEI Titan Themis scanning transmission electron microscope (at 80 kV) and a Hitachi S-4700 cold field emission scanning electron microscope. The surface chemical states of the samples were characterized with a PHI 5800 X-ray photoelectron spectrometer. The light absorption properties of the samples were determined with a Shimadzu UV-2450 UV‒visible spectrometer and a Bomem MB-160 near-infrared spectrometer. The time-resolved fluorescence decay curves of the samples were collected on a Horiba iHR320 spectrometer with a Spectra-Physics 400 nm ion laser (150 fs laser pulse excitation). The molecular vibrations of the samples were recorded on a Shimadzu IRTracer-100 Fourier transform infrared spectrometer with an attenuated total reflectance accessory, while the temperature-variable diffuse reflectance infrared Fourier transform spectroscopy (DRIFTS) measurements were carried out on a Shimadzu IRAffinity-1 spectrometer equipped with an in-situ PIKE Technologies DiffusIR cell. The thermal decomposition processes of the samples were investigated with a Mettler Toledo TGA/SDTA851e thermogravimetric analyzer under a 10 mL min^−1^ air flow. The structure of the AMM film was assessed via small-angle X-ray scattering (SAXS) measurements done at the 12-ID-C station of the Advanced Photon Source at Argonne National Laboratory with an X-ray energy of 18.0 keV (0.1 s exposure time) and a Pilatus2M detector located approximately 2.2 m downstream of the sample.

### Electrochemical tests

Electrochemical tests were carried out in a three-electrode cell in which a fluorine-doped tin oxide glass coated with sample, Pt, and a saturated calomel electrode acted as the working, counter, and reference electrodes, respectively. A 0.5 M sodium sulfate aqueous solution was used as the electrolyte. Mott-Schottky analyses were conducted at 1, 2, and 3 kHz in the dark, while the transient photocurrent response was acquired under visible light irradiation (λ > 400 nm, 0.85 kW m^−2^).

### Mechanical tests

All film samples (except the original leaf) subjected to the mechanical tests were prepared by wet casting (immediately after synthesis) and subsequent drying at room temperature. The mechanical tests were conducted on a TA Instrument Q800 dynamic mechanical analyzer. The tensile properties of the samples were determined at room temperature and at a constant speed of 3 N min^−1^ until fracture. The effects of temperature on the tensile properties of the AMM were further investigated at 100 °C in the same manner. Dynamic mechanical analyses were carried out for the AMM and the original leaf in the temperature range of 30-190 °C with the frequency and amplitude set as 2 Hz and 5 μm (0.03% of the sample length), respectively.

### Biodegradability tests

To evaluate the biodegradabilities, thin films of the AMM and daily used polyethylene plastics (Ziplock bag, shopping bag, and plastic wrap) were buried in natural soil at a depth of 10 cm. The samples were monitored periodically to assess the biodegradation degrees.

### Solar water evaporation

To test the solar water evaporation performance, an AMM film (1 g, 3 cm × 3 cm) was placed in a 100 mL beaker filled with 20 mL of deionized water and placed under simulated sunlight irradiation provided by a Newport 300 W xenon lamp equipped with an AM1.5 G filter. A Sartorius Entris analytical balance with a 0.1 mg accuracy was utilized to measure the evaporation rate in real time. An Omega HH11 digital thermometer was used to measure the surface temperature of the AMM film. The solar-to-steam conversion efficiency was calculated via Eq. 4.

4 $${{{{{\rm{\eta }}}}}}=\frac{{{{{{\rm{v}}}}}}\times {{{{{{\rm{H}}}}}}}_{{{{{{\rm{e}}}}}}}}{{{{{{\rm{I}}}}}}}\times 100\%$$

in which v, H_e_, and I represent the evaporation rate (per area), the heat of water evaporation (~2260 kJ kg^−1^), and the light intensity, respectively.

### Photocatalytic hydrogen production

An AMM film (40 mg, 1.5 cm × 1.5 cm) was placed obliquely in a quartz reactor with 10 mL of a 30 vol% methanol solution. The reactor was evacuated with a vacuum pump to remove impurity gases. Then, the AMM film was illuminated by a Newport 300 W xenon lamp with an AM1.5 G filter operated at 1 kW m^−2^ or with both an AM1.5 G and a UVcut400 filter operated at 0.85 kW m^−2^. The gas products were measured with a 3D Instruments Accu-Cal Plus pressure gauge and a Hewlett Packard 5890 Series II gas chromatograph equipped with a Porapak Q column and a thermal conductivity detector. The as-generated formaldehyde in the aqueous solution was analyzed via acetylacetone spectrophotometry at 413 nm with a Shimadzu UV-2450 UV‒visible spectrometer. The cycling tests were conducted by simply flushing the surface of the AMM film with deionized water after each cycle.

For comparison, films of the lignin-cellulose, lignin, cellulose, and the original leaf, as well as the pressed disks (at 200 MPa) of whewellite, hydrochar, pyrochar, titanium dioxide (TiO_2_), and 1 wt% Pt/TiO_2_ (prepared via impregnation of TiO_2_ in an aqueous solution of chloroplatinic acid hexahydrate and subsequent calcination and reduction) with the same weight and area, were also tested to determine photocatalytic hydrogen production.

### Photocatalytic degradation of tetracycline

An AMM film (1 g, 3 cm × 3 cm) was placed in a 100 mL beaker that contained 50 mL of a 10 mg L^−1^ tetracycline aqueous solution. Prior to the photocatalytic reaction, the beaker was left in the dark for 30 min to reach adsorption equilibrium. Afterward, light was introduced from the top with a Newport 300 W xenon lamp. Simulated sunlight was provided with an AM1.5 G filter operating at 1 kW m^−2^, and visible light (λ > 400 nm, 0.85 kW m^−2^) was provided by overlying a UVcut400 filter onto the AM1.5 G filter. The photocatalytic reaction lasted for 2 h, during which 3 mL of the solution was collected at intervals of 30 min for analysis with a Shimadzu UV-2450 UV‒visible spectrometer after filtration through a 0.22 μm membrane. The cycling tests were conducted by simply flushing the surface of the AMM film with deionized water after each cycle.

For comparison, films of the lignin-cellulose, lignin, cellulose, and the original leaf, as well as the pressed disks (at 200 MPa) of whewellite, hydrochar, pyrochar, titanium dioxide (TiO_2_), zinc oxide (ZnO), tungsten oxide (WO_3_), cerium oxide (CeO_2_), zirconium oxide (ZrO_2_), gallium oxide (Ga_2_O_3_), molybdenum sulfide (MoS_2_), and tungsten sulfide (WS_2_) with the same weight (1 g), were also tested for visible-light photocatalytic degradation of tetracycline. The efficiencies for removal of the tetracycline by adsorption (R_a_) and photocatalysis (R_p_) were defined as follows:

5 $${{{{{{\rm{R}}}}}}}_{{{{{{\rm{a}}}}}}}=\left(1-\frac{{{{{{{\rm{C}}}}}}}_{{{{{{\rm{ad}}}}}}}}{{{{{{{\rm{C}}}}}}}_{0}}\right)\times 100\%$$

6 $${{{{{{\rm{R}}}}}}}_{{{{{{\rm{p}}}}}}}=\left(1-\frac{{{{{{\rm{C}}}}}}}{{{{{{{\rm{C}}}}}}}_{{{{{{\rm{ad}}}}}}}}\right)\times 100\%$$

where C_0_, C_ad_, and C (mg L^−1^) are the initial, post-adsorption (for 30 min), and real-time (at t min) concentrations of tetracycline, respectively. The rate constant (k) was acquired by fitting the experimental data with the pseudo-first order kinetic model (Eq. 7).

7 $${{{{{\rm{ln}}}}}} \, {{{{{\rm{C}}}}}}={{{{{\rm{ln}}}}}} \, {{{{{{\rm{C}}}}}}}_{{{{{{\rm{ad}}}}}}}-{{{{{\rm{kt}}}}}}$$

The products generated in the visible-light photocatalytic degradation of tetracycline over the AMM film were analyzed with a Thermo Scientific LCQ Fleet MS system, and their structures were drawn with ChemDraw software. The toxicities of these products, in terms of the fathead minnow 50% lethal concentration (96 h), developmental toxicity, and mutagenicity, were assessed by using the Toxicity Estimation Software Tool developed by the United States Environmental Protection Agency.

### Density functional theory (DFT) calculations

Lignin was modeled as guaiacyl glycerol-β-guaiacyl ether, which is a β-O-4 dimer representing the building blocks of natural lignin^51^. Cellulose was modeled with cellobiose, which is a glucose dimer containing a β-O-4 glycosidic bond^52^. Four units of whewellite were retrieved from the whewellite monoclinic structure for use as the model for calculations^53^. The structures of lignin, cellulose, whewellite, lignin-cellulose composite, and lignin-cellulose-whewellite composite were optimized by employing the PM6 Hamiltonian with Grimme’s D3 dispersion correction implemented in the Gaussian 16 package^54^. The M062X hybrid exchange-correlation functional with the 6-31 + G(d,p) basis set and Grimme’s D3 dispersion correction were used for the energy calculations, since they have been found to be effective in describing intermolecular interactions^51^. The binding energy of the composite (E_bind_) was obtained via Eq. 8.

8 $${{{{{{\rm{E}}}}}}}_{{{{{{\rm{bind}}}}}}}={{{{{\rm{E}}}}}}-{{{{{{\rm{E}}}}}}}_{1}-{{{{{{\rm{E}}}}}}}_{2}-{{{{{{\rm{E}}}}}}}_{3}$$

in which E is the energy of the composite, and E_1_, E_2_, and E_3_ represent the energy of each constituent. Moreover, the electrostatic potential and electron density difference analyses were conducted with Multiwfn 3.8 (dev) code^55^ and visualized via Visual Molecular Dynamics software^56^.

In addition, the redox potential for tetracycline hydroxylation (Eq. 9) was calculated based on the standard-state Gibbs free energy of gas-phase molecule (G_gas_, via CBS-4M), the energy of the gas-phase molecule (E_gas_, via M052X/6-31 G(d)), and the energy of the molecule dissolved in water (E_dis_, via M052X/6-31 G(d) with the solvation model of water based on density). Namely, the aqueous Gibbs free energy (G_aq_) of each species in Eq. 9 was derived via Eq. 10, according to which the aqueous Gibbs free energy change of the reaction (ΔG_aq_) was acquired^57^. Furthermore, the redox potential was calculated with the Nernst equation and referenced to the standard hydrogen electrode (Eq. 11)^57^.

9 $${{{{{\rm{T}}}}}}{{{{{\rm{etracycline}}}}}}+{{{{{{\rm{H}}}}}}}_{2}{{{{{\rm{O}}}}}}={{{{{\rm{Hydroxylated\; t}}}}}}{{{{{\rm{etracycline}}}}}}+2{{{{{{\rm{H}}}}}}}^{+}+2{{{{{{\rm{e}}}}}}}^{-}$$

10 $${{{{{{\rm{G}}}}}}}_{{{{{{\rm{aq}}}}}}}={{{{{{\rm{G}}}}}}}_{{{{{{\rm{gas}}}}}}}+({{{{{{\rm{E}}}}}}}_{{{{{{\rm{gas}}}}}}}-{{{{{{\rm{E}}}}}}}_{{{{{{\rm{dis}}}}}}})$$

11 $${{{{{\rm{E}}}}}}=\frac{{\triangle {{{{{\rm{G}}}}}}}_{{{{{{\rm{aq}}}}}}}}{{{{{{\rm{nF}}}}}}}-4.28$$

## Supplementary information

Supplementary Information

Description of Additional Supplementary Files

Supplementary Data 1

Supplementary Data 2

## Data Availability

Source data are provided with this paper.

## Source data

Source Data
