# Supplementary material for: Turning dead leaves into an active multifunctional material as evaporator, photocatalyst, and bioplastic
Source: Nat Commun. 2023 Mar 2;14:1203. doi: 10.1038/s41467-023-36783-8 (PMC9981597; doi:10.1038/s41467-023-36783-8)
Supplement: Supplementary file 1 — Supplementary Information [file 41467_2023_36783_MOESM1_ESM.pdf]

Supplementary Information

Turning dead leaves into an active multifunctional material as evaporator, photocatalyst, and bioplastic

Siyuan Fang[1], Xingyi Lyu[2], Tian Tong[3], Aniqa Ibnat Lim[3], Tao Li[2,4], Jiming Bao[3], Yun Hang Hu[1]*

[1]Department of Materials Science and Engineering, Michigan Technological University, Houghton, Michigan 49931, United States

[2]Department of Chemistry and Biochemistry, Northern Illinois University, DeKalb, Illinois 60115, United States

[3]Department of Electrical and Computer Engineering, University of Houston, Houston, Texas 77204, United States

[4]X-ray Science Division, Argonne National Laboratory, Lemont, Illinois 60439, United States

*Corresponding author. Email: yunhangh@mtu.edu

Contents

1. Adjustable thickness of AMM film 4

2. Chemical composition of AMM and original leaf.. 5

3. X-ray photoelectron spectra of AMM and original leaf powders 6

4. Optimized molecular structure, electrostatic potential maps, and electron density difference plots of lignin-cellulose-whewellite composite 8

5. Optimized molecular structure and electrostatic potential maps of lignin-cellulose composite 9

6. Scanning electron microscopy images of original leaf 10

7. Fourier transform near-infrared spectra..11

8. Ultraviolet-visible spectra of AMM without pigments or minerals 12

9. Solar water evaporation efficiency of AMM and other control samples...........13

10. Photocatalytic activity of AMM without pigments 14

11. Band structure determination 15

12. Measured temperatures and dark control experiments for hydrogen production 18

13. Cycling performance of photocatalytic hydrogen production over AMM film 19

14. Visible-light photocatalytic degradation pathway of tetracycline over AMM ..20

15. Toxicity evaluation of products in visible-light photocatalytic degradation of tetracycline over AMM 21

16. Tensile strength of AMM in comparison with petroleum-based plastics 22

17. Mechanical properties of AMM and other control samples 23

18. Temperature-dependent damping factor of AMM and original leaf 24

19. Differential thermogravimetric curves of AMM and original leaf....................25

20. *In-situ* diffuse reflectance infrared Fourier transform spectra of AMM...........26

21. Life cycle assessment 27

22. AMM prepared from another dead leaves..28

23. AMM prepared from the leaf pulp and leaf vein of red maple 29

24. Comparison with traditional technologies for treating dead leaves in terms of carbon emission 30

25. Characterizations of control samples 32

Supplementary references..33

1. Adjustable thickness of AMM film

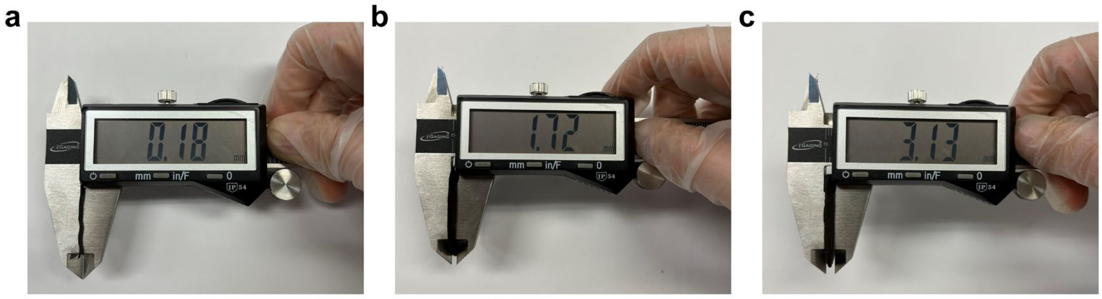

Supplementary Fig. 1: Adjustable thickness of AMM film. a 0.18 mm, **b** 1.72 mm, and **c** 3.13 mm.

2. Chemical composition of AMM and original leaf

Supplementary Table 1: Chemical composition of AMM and original leaf.

Component	AMM	Original leaf
Lignin (%)	36.61	29.83
Cellulose (%)	44.13	35.29
Hemicellulose (%)	2.71	24.03
Whewellite (%)	11.01	4.19
Chlorophylls (%)	0.00	0.00
Carotenoids (%)	0.00	0.01
Flavonoids (%)	0.56	1.70
Anthocyanins (%)	0.02	0.03
K (%)	0.01	0.09
Ca (%)	3.02	1.15
Na (%)	0.01	0.02
Mg (%)	0.05	0.09
Fe (%)	0.02	0.02
Mn (%)	0.01	0.02

Notes: The amount of each component was obtained by conducting three parallel experiments and expressed as its average proportion in the total dry weight (%).

3. X-ray photoelectron spectra of AMM and original leaf powders

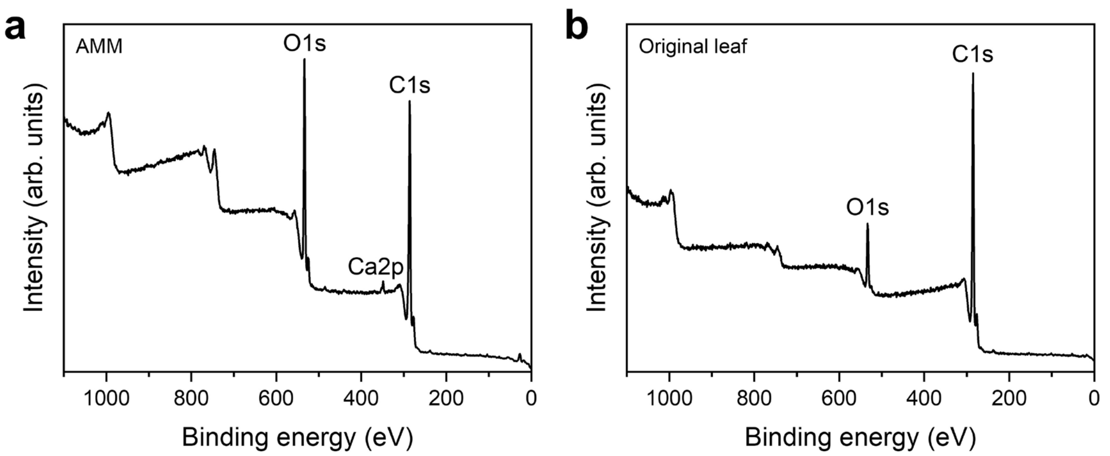

Supplementary Fig. 2: XPS survey scan of powdered samples. a AMM. **b** Original leaf.

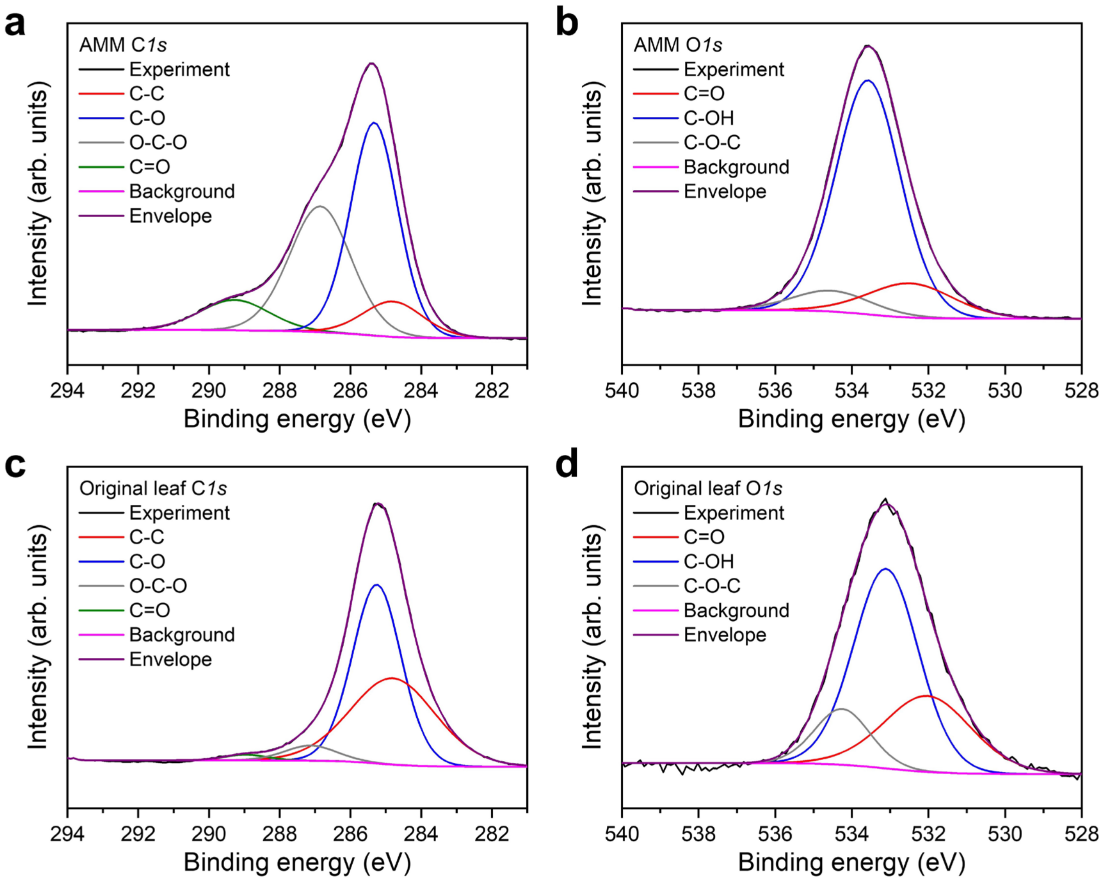

Supplementary Fig. 3: XPS fine spectra of powdered samples. a C*1s* and **b** O*1s* XPS

spectra of AMM. **c** C*1s* and **d** O*1s* XPS spectra of original leaf.

Supplementary Table 2: Elemental composition of AMM and original leaf obtained from XPS spectra.

**Element**	**AMM**	**Original leaf**
C (wt%)	75.7	85.4
O (wt%)	22.0	14.6
Ca (wt%)	2.3	0

4. Optimized molecular structure, electrostatic potential maps, and electron density difference plots of lignin-cellulose-whewellite composite

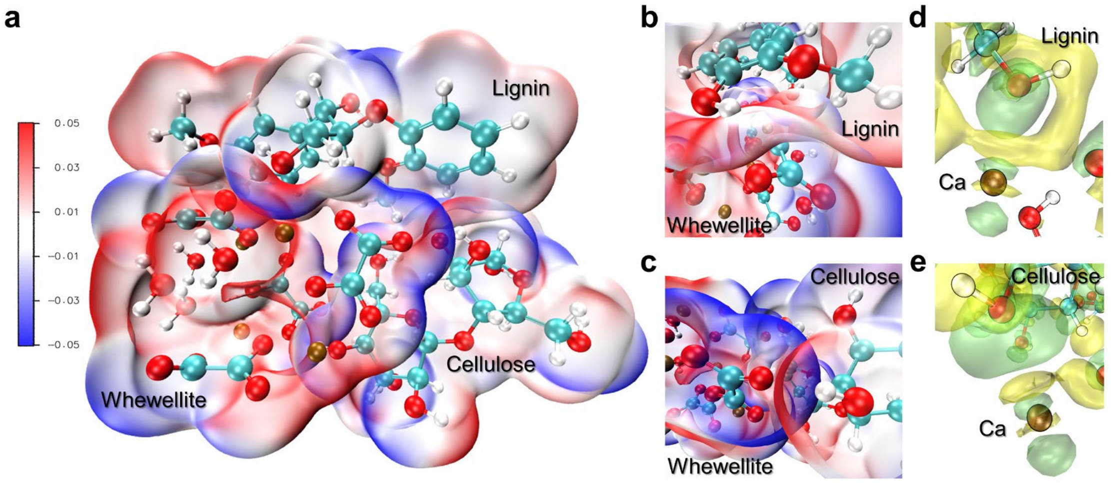

Supplementary Fig. 4: Structure and interaction analysis of lignin-cellulose-whewellite composite based on theoretical calculations. a Optimized molecular structure and electrostatic potential maps and close-up views of **b** the lignin-whewellite interactions and **c** the cellulose-whewellite interactions. **d**, **e** Electron density difference plots after forming the lignin-cellulose-whewellite composite from the single components; the yellow and green regions represent electron acquisition and depletion, respectively. The carbon, oxygen, hydrogen, and calcium atoms are marked in cyan, red, white, and brown, respectively.

5. Optimized molecular structure and electrostatic potential maps of lignin-cellulose composite

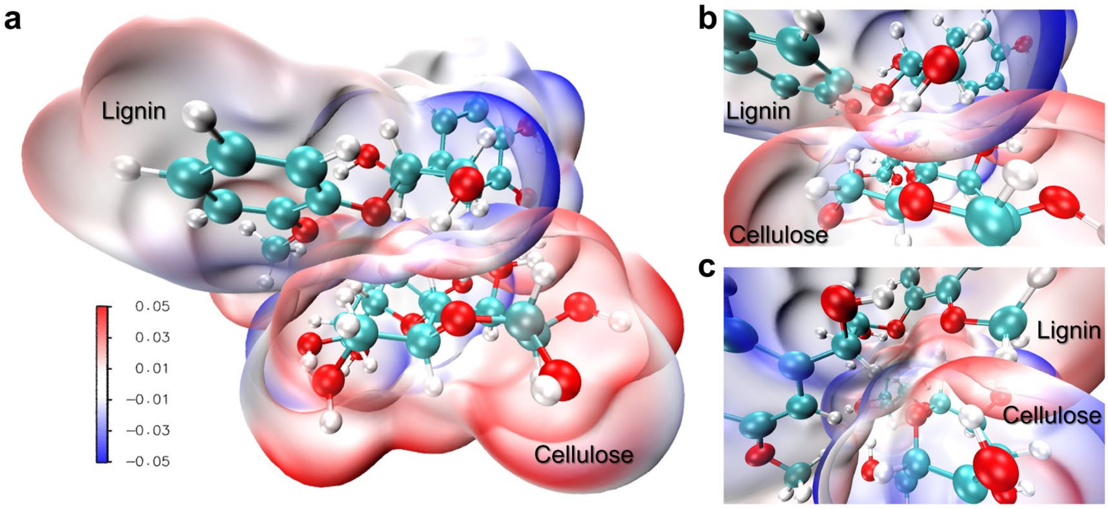

Supplementary Fig. 5: Optimized molecular structure and electrostatic potential maps of lignin-cellulose composite based on theoretical calculations. a Overview. **b c** Close-up views. The carbon, oxygen, and hydrogen atoms are marked in cyan, red, and white, respectively.

6. Scanning electron microscopy images of original leaf

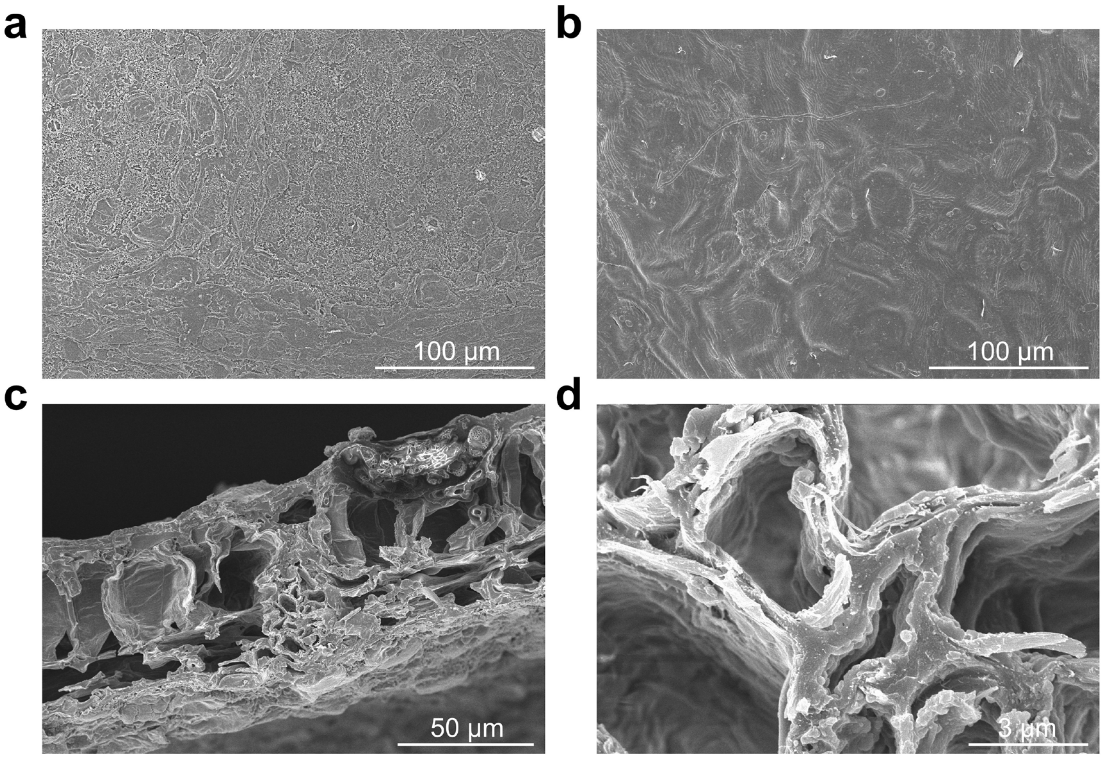

Supplementary Fig. 6: Scanning electron microscopy images of original leaf. a, b Top-view images of two sides. **c**, **d** Cross-sectional images.

7. Fourier transform near-infrared spectra

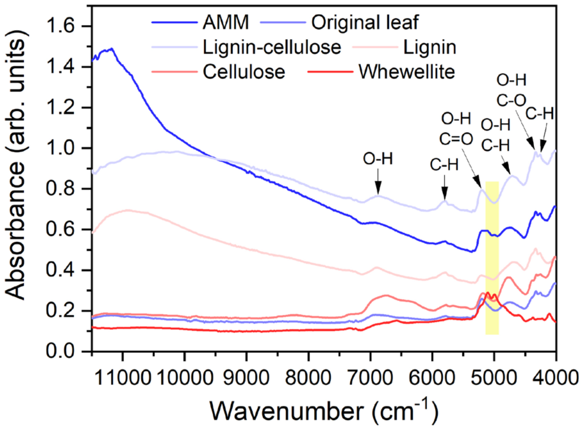

Supplementary Fig. 7: Fourier transform near-infrared spectra.

8. Ultraviolet-visible spectra of AMM without pigments or minerals

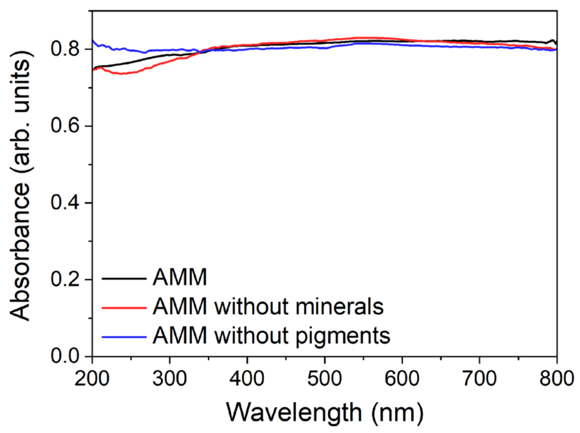

Supplementary Fig. 8: Ultraviolet-visible spectra of AMM without pigments or minerals in comparison with that of AMM.

9. Solar water evaporation efficiency of AMM and other control samples

Supplementary Table 3: Solar water evaporation efficiency at 1 kW m^{-2}.

Material	Water evaporation rate (kg m^{-2} h^{-1})	Efficiency (%)
AMM	0.84	52.5
AMM without pigments	0.84	52.5
Lignin-cellulose	0.83	52.2
Lignin	0.74	46.6
Cellulose	0.59	37.2
None	0.55	34.6

10. Photocatalytic activity of AMM without pigments

Supplementary Table 4: Photocatalytic activity of AMM without pigments in comparison with that of AMM under simulated sunlight irradiation.

Photocatalytic process	Material	Activity
Hydrogen production	AMM without pigments	12.0 μmol H_2 h^{-1} cm^{-2}
	AMM	12.4 μmol H_2 h^{-1} cm^{-2}
Degradation of tetracycline	AMM without pigments	97.0% removal
	AMM	96.7% removal

11. Band structure determination

The band structures of lignin, cellulose, and whewellite were determined by Mott-Schottky curves, valence-band X-ray photoelectron spectra, and Tauc plots. Firstly, Mott-Schottky curves (Supplementary Fig. 9) reveal the Fermi level of -0.71, -0.35, and -0.45 V vs. saturated calomel electrode (SCE) for lignin, cellulose, and whewellite, respectively. These values are further converted to -0.47, -0.11, and -0.21 V vs. standard hydrogen electrode (SHE), respectively. Then the valence-band X-ray photoelectron spectra (Supplementary Fig. 10) were collected, suggesting the absorption onset at 0.51, 2.25, and 0.13 eV for lignin, cellulose, and whewellite, respectively. Since the origin point of X-ray photoelectron spectra represents Fermi level, the valence band top is then estimated as 0.04, 2.14, and -0.08 V vs. SHE for lignin, cellulose, and whewellite, respectively. Furthermore, Tauc plots (Supplementary Fig. 11) derived from ultraviolet-visible spectra (Supplementary Fig. 12) and Fourier transform near-infrared spectra (Supplementary Fig. 7) demonstrate the band gap of 1.08, 3.12, and 2.96 eV for lignin, cellulose, and whewellite, respectively, according to which the conduction band bottom is calculated as -1.04, -0.98, and -3.04 V vs. SHE, respectively. These data are summarized in Supplementary Table 5.

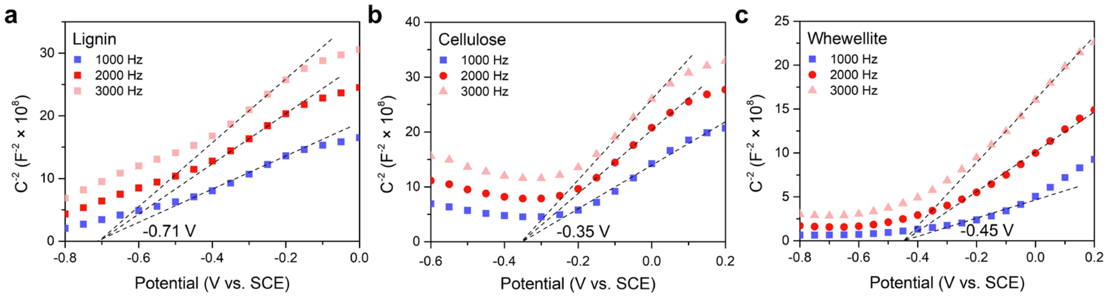

Supplementary Fig. 9: Mott-Schottky curves. a Lignin. **b** Cellulose. **c** Whewellite.

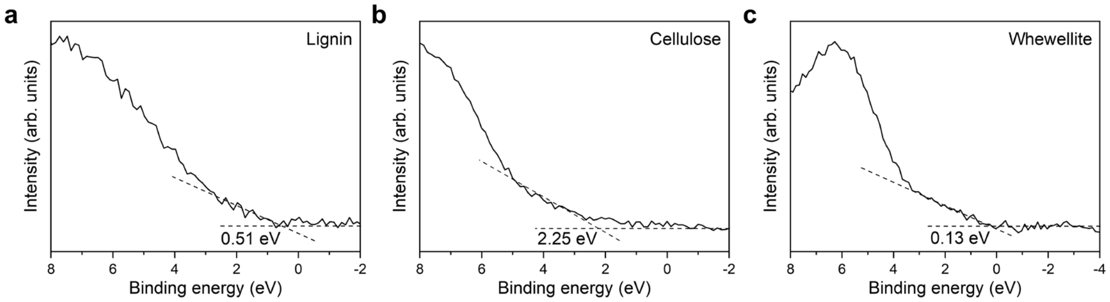

Supplementary Fig. 10: Valence-band X-ray photoelectron spectra. a Lignin. **b** Cellulose. **c** Whewellite.

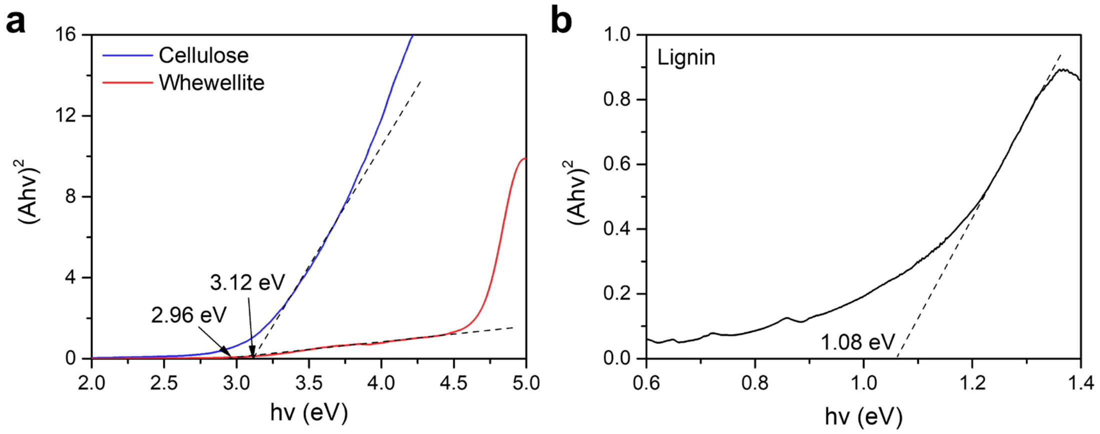

Supplementary Fig. 11: Tauc plots. a Tauc plots derived from ultraviolet-visible spectra for cellulose and whewellite. **b** Tauc plot derived from Fourier transform near-infrared spectrum for lignin.

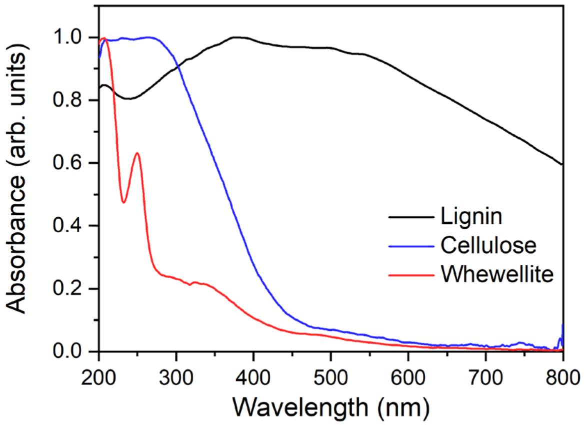

Supplementary Fig. 12: Ultraviolet-visible spectra (after normalization).

Supplementary Table 5: Band structure of lignin, cellulose, and whewellite.

Parameter	Lignin	Cellulose	Whewellite
Fermi level (V vs. SCE)	-0.71	-0.35	-0.45
Fermi level (V vs. SHE)	-0.47	-0.11	-0.21
Onset of valence-band XPS spectrum (eV)	0.51	2.25	0.13
Valence band top (V vs. SHE)	0.04	2.14	-0.08
Band gap (eV)	1.08	3.12	2.96
Conduction band bottom (V vs. SHE)	-1.04	-0.98	-3.04

12. Measured temperatures and dark control experiments for hydrogen production

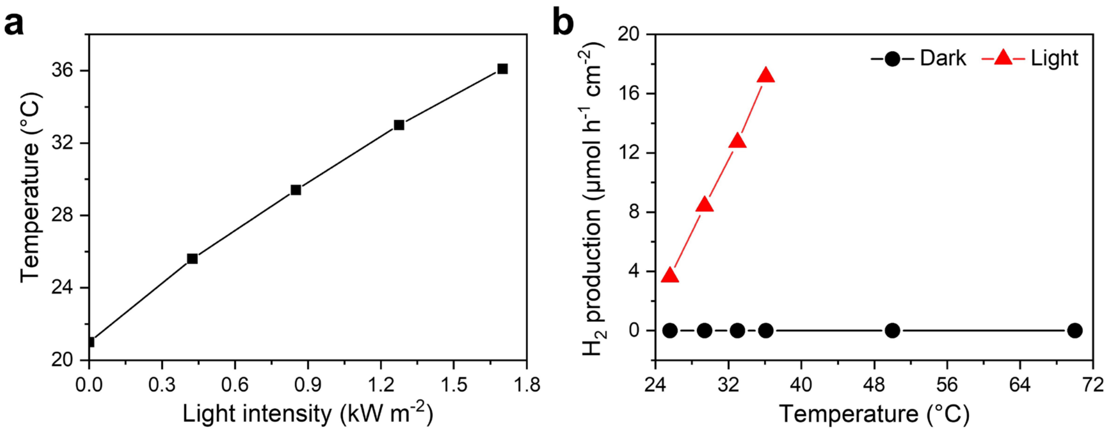

Supplementary Fig. 13: Measured temperatures and dark control experiments for hydrogen production. a Temperature of AMM surface in methanol-water mixture under visible light irradiation at various light intensities. **b** Hydrogen production in dark control experiments (with external resistive heating) in comparison with the photocatalytic tests.

13. Cycling performance of photocatalytic hydrogen production over AMM film

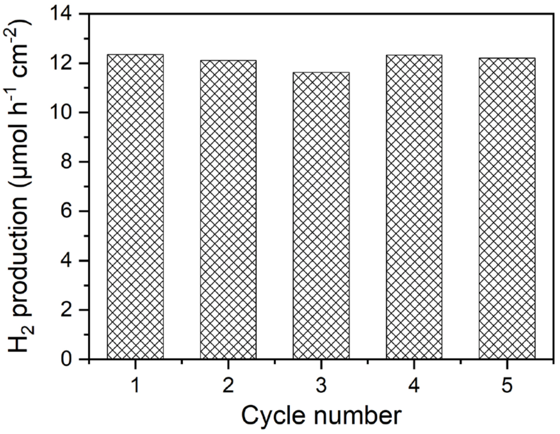

Supplementary Fig. 14: Cycling performance of photocatalytic hydrogen production over AMM film under simulated sunlight (1 kW m^{-2}).

14. Visible-light photocatalytic degradation pathway of tetracycline over AMM

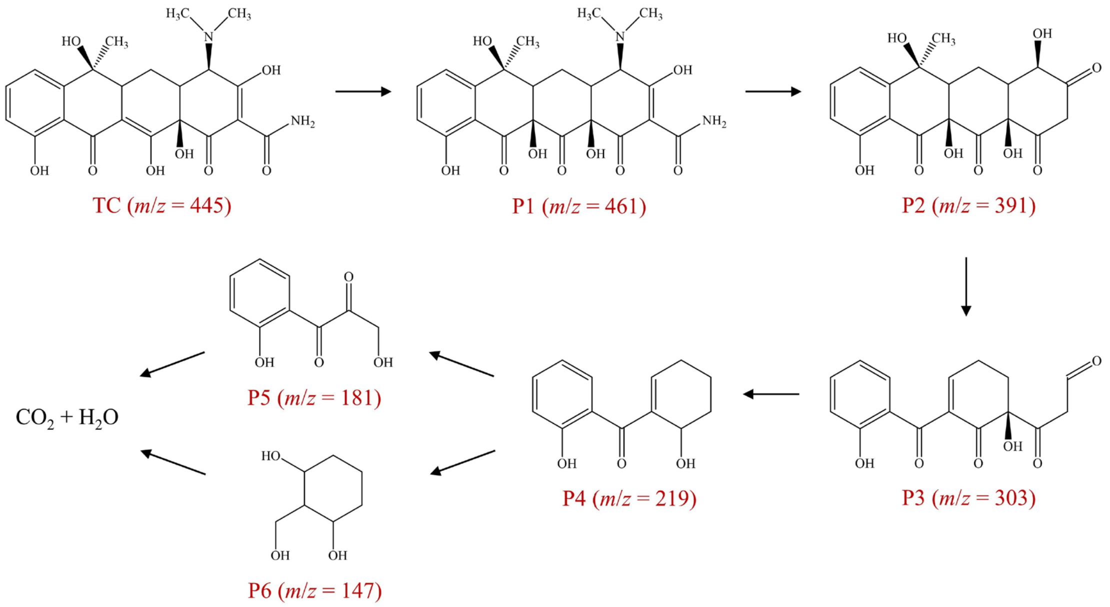

Supplementary Fig. 15: Visible-light photocatalytic degradation pathway of tetracycline (TC) over AMM.

15. Toxicity evaluation of products in visible-light photocatalytic degradation of tetracycline over AMM

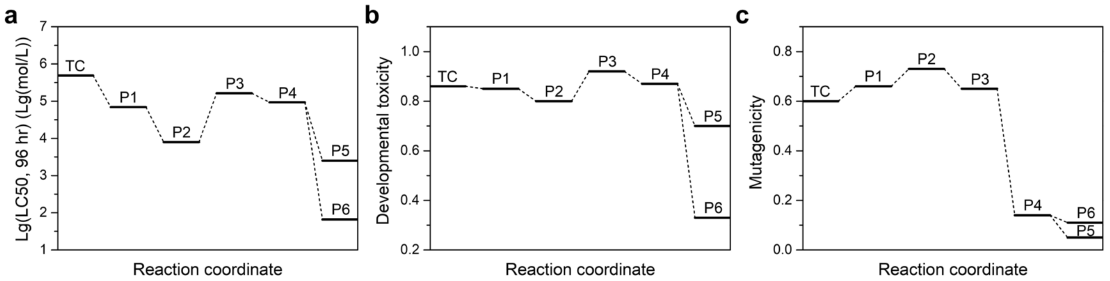

Supplementary Fig. 16: Toxicity evaluation of products in visible-light photocatalytic degradation of tetracycline (TC) over AMM. a Acute toxicity based on the fathead minnow 50% lethal concentration (96 h). **b** Developmental toxicity. **c** Mutagenicity.

16. Tensile strength of AMM in comparison with petroleum-based plastics

Supplementary Table 6: Tensile strength of AMM in comparison with petroleum-based plastics.

Material	Tensile strength (MPa)	Reference
AMM	132.0	This work
Acrylonitrile butadiene styrene (ABS)	33.7	[1]
High-density polyethylene (HDPE)	20.7	[2]
Polyamide (PA)	45.0	[3]
Polycarbonate (PC)	71.8	[3]
Polyethylene (PE)	39.7	[3]
Polyethylene terephthalate (PET)	54.8	[3]
Polymethyl methacrylate (PMMA)	73.7	[3]
Polypropylene (PP)	35.1	[4]
Polystyrene (PS)	23.9	[5]
Polyvinyl chloride (PVC)	42.3	[6]
Polyvinyl alcohol (PVA)	72.2	[7]
Polylactide (PLA)	48.7	[8]
Polybutylene adipate co-terephthalate (PBAT)	11.0	[8]
Polyhydroxyalkanoate (PHA)	22.4	[9]

17. Mechanical properties of AMM and other control samples

Supplementary Table 7: Mechanical properties of AMM and other control samples.

Material	Tensile strength (MPa)	Young's modulus (MPa)	Toughness (kJ m^{-3})
Original leaf	1.1	4.6	60.7
Lignin-cellulose	3.4	1987.7	24.2
Lignin	2.4	1072.2	12.2
Cellulose	1.1	372.9	3.8
AMM	132.0	50401.9	344.0
AMM at 100 °C	117.4	39004.0	686.1

18. Temperature-dependent damping factor of AMM and original leaf

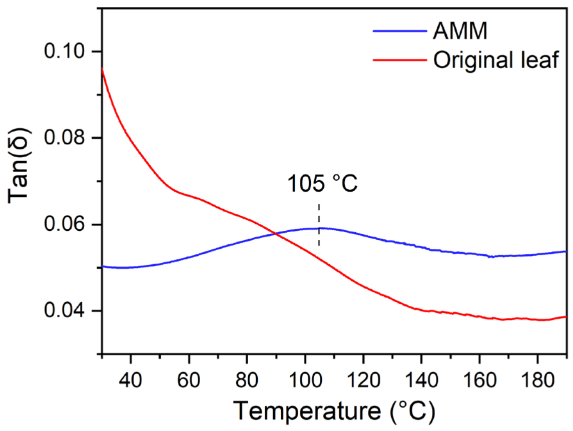

Supplementary Fig. 17: Temperature-dependent damping factor of AMM and original leaf.

19. Differential thermogravimetric curves of AMM and original leaf

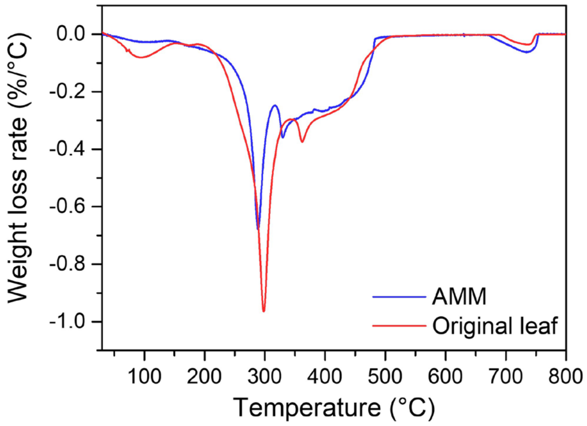

Supplementary Fig. 18: Differential thermogravimetric curve of AMM and original leaf.

20. *In-situ* diffuse reflectance infrared Fourier transform spectra of AMM

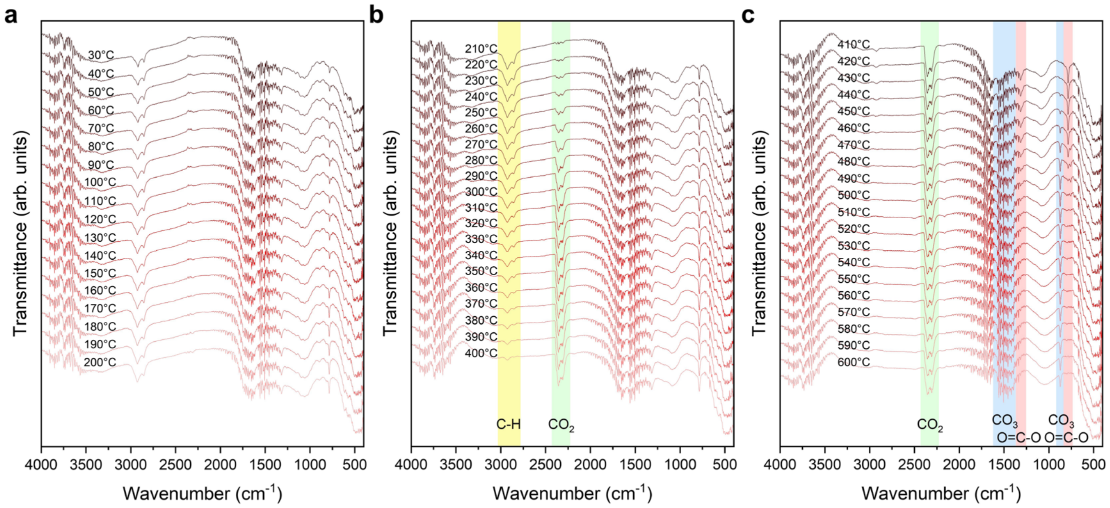

Supplementary Fig. 19: *In-situ* diffuse reflectance infrared Fourier transform spectra of AMM at elevated temperatures in air. a 30-200 °C. **b** 210-400 °C. **c** 410-600 °C.

21. Life cycle assessment

Supplementary Table 8: Life cycle assessment of AMM in comparison with acrylonitrile butadiene styrene (ABS), polyvinyl fluoride (PVF), and wood-derived bioplastic (WBP)[10].

Environmental impact	ABS	PVF	WBP	AMM
Acidification (10^{-7} kg SO_2 cm^{-3} MPa^{-1})	3.9~13.8	17.9~36.8	5.2~6.9	5.0~6.6
Ecotoxicity (10^{-4} CTU cm^{-3} MPa^{-1})	3.3~11.9	14.2~29.0	4.8~7.1	4.6~6.9
Eutrophication (10^{-7} kg N cm^{-3} MPa^{-1})	3.7~13.6	11.8~24.3	5.7~8.6	5.6~8.3
Global warming potential (10^{-5} kg CO_2 cm^{-3} MPa^{-1})	12.3~43.6	34.1~69.9	13.1~17.1	12.7~16.5
Ozone depletion (10^{-12} kg CFC cm^{-3} MPa^{-1})	5.5~18.7	29.4~60.4	12.1~17.6	11.7~17.0
Smog formation (10^{-6} kg O_3 cm^{-3} MPa^{-1})	4.1~14.3	18.9~38.8	5.4~7.1	5.2~6.9
Fossil fuel depletion (10^{-4} MJ surplus cm^{-3} MPa^{-1})	2.5~8.9	3.7~7.6	3.1~3.5	3.1~3.4
Respiratory effects (10^{-8} kg $PM_{2.5}$ cm^{-3} MPa^{-1})	14.4~49.8	45.4~92.8	21.3~32.0	20.7~31.0
Human health – carcinogenic (10^{-12} CTU cm^{-3} MPa^{-1})	7.4~26.3	15.0~30.6	6.7~9.9	6.5~9.6
Human health - non-carcinogenic (10^{-11} CTU cm^{-3} MPa^{-1})	1.2~4.2	6.5~13.2	2.0~2.9	1.9~2.8

Notes: CTU is comparative toxicity unit. CFC-11 represents trichlorofluoromethane. $PM_{2.5}$ means particulate matters with a diameter below 2.5 μm.

22. AMM prepared from another dead leaves

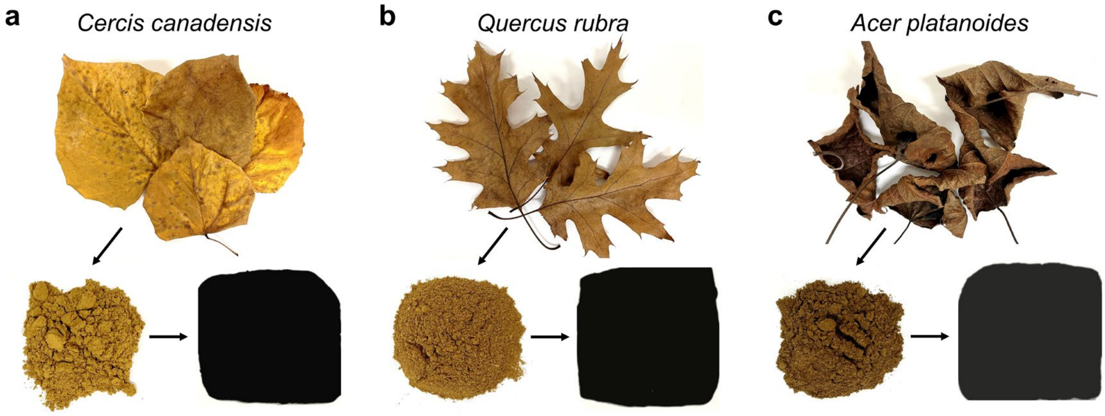

Supplementary Fig. 20: Preparation of AMM film from another dead leaves. a *Cercis canadensis*, **b** *Quercus rubra*, and **c** *Acer platanoides*.

Supplementary Table 9: Performance of AMM films prepared from various dead leaves.

**Raw material**	**Tensile strength (MPa)**	**Solar-to-steam efficiency (%)**	**H_2 production rate (μmol h^{-1} cm^{-2})**	**Tetracycline removal efficiency (%)**
Cercis canadensis	147.2	50.7	11.4	92.3
Quercus rubra	120.5	56.0	11.8	97.1
Acer platanoides	139.7	52.2	12.7	96.0
Red maple	132.0	52.5	12.4	96.7

Notes: Simulated sunlight at 1 kW m^{-2} was used for solar water evaporation, photocatalytic hydrogen production, and photocatalytic degradation of tetracycline.

23. AMM prepared from the leaf pulp and leaf vein of red maple

Supplementary Table 10: Performance of AMM films prepared from the leaf pulp and leaf vein of red maple.

Raw material	Tensile strength (MPa)	Solar-to-steam efficiency (%)	H_2 production rate (μmol h^{-1} cm^{-2})	Tetracycline removal efficiency (%)
Leaf pulp	119.7	50.5	11.9	97.3
Leaf vein	125.2	55.7	10.5	86.9
Entire leaf	132.0	52.5	12.4	96.7

Notes: Simulated sunlight at 1 kW m^{-2} was used for solar water evaporation, photocatalytic hydrogen production, and photocatalytic degradation of tetracycline.

24. Comparison with traditional technologies for treating dead leaves in terms of carbon emission

Supplementary Table 11: Carbon emissions of various technologies for treating dead leaves.

Carbon emission (ton CO_2/ton leaves)	Incineration[11]	Landfill[11]	Composting[11]	This work
CO_2 emission in treatment	2.0	3.8	1.7	20.4
Biogenic CO_2 uptake	-1.5	-1.5	-1.5	-1.5
Electricity substitution	-0.8			
Fertilizer substitution			0.0	
Plastic substitution (ABS)				-19.9[10]
Water evaporation material substitution (graphene)				-46.1[12]
Photocatalyst substitution for antibiotics removal (TiO_2)				-35.0[13]
Photocatalyst substitution for H_2 production (1% Pt/TiO_2)				-37.7[13,14]
Total CO_2 emission	-0.3	2.3	0.2	-119.7

Notes: (1) The CO_2 emissions of incineration, landfill, and composting are derived from a previous paper[11]. (2) The CO_2 emission in the process of turning dead leaves into AMM covers the upstream production of chemicals, transportation, electricity, water, labor, etc. (3) The reduced CO_2 emissions by substituting the plastic (ABS), water evaporation material (graphene), photocatalyst for antibiotics removal (TiO_2), and photocatalyst for H_2 production (1% Pt/TiO_2) are calculated via Eqn. S1:

$$E_e = E_S \times \frac{P_{AMM}}{P_S} \times Y_{AMM} \quad (S1)$$

where E_e, E_S, P_S, P_{AMM}, and Y_{AMM} are the equivalent CO_2 emission (ton CO_2/ton leaves), CO_2 emission of producing the substituted material (ton CO_2/ton) (obtained from literature), performance of the substituted material, performance of AMM, and yield of

AMM (57.7%), respectively. The tensile strength, solar-to-steam efficiency, tetracycline removal efficiency, and H_2 production rate (under 1 kW m^{-2} simulated sunlight), are used as the indicator of the performance.

25. Characterizations of control samples

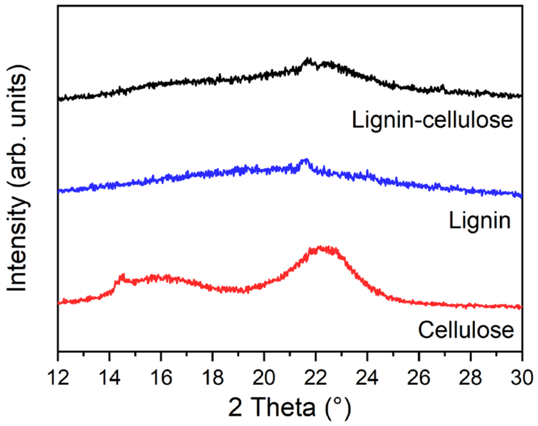

Supplementary Fig. 21: X-ray diffraction patterns of control samples.

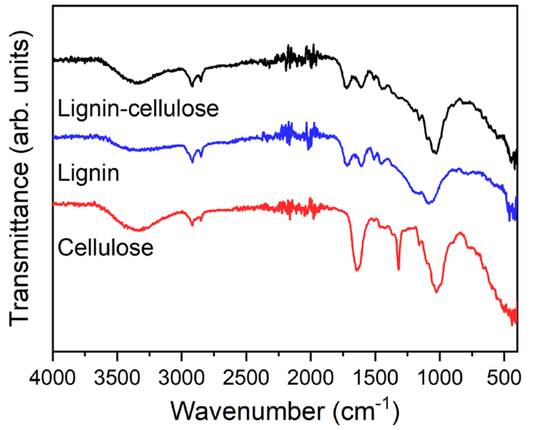

Supplementary Fig. 22: Fourier transform infrared spectra of control samples.

Supplementary references

1 Prapul Chandra, A. C., Byregowda, H. V., Gangadhar, T. G., Angadi, G. & Krishna, M. Investigation the effect of electroplating and filler material on the tensile properties of wollastonite reinforced ABS composites. *J. Phys. Conf. Ser.* **1240**, 012105 (2019).

2 Mahmoud, M. E. *et al.* Design and testing of high-density polyethylene nanocomposites filled with lead oxide micro- and nano-particles: Mechanical, thermal, and morphological properties. *J. Appl. Polym. Sci.* **136**, 47812 (2019).

3 Zhang, K. *et al.* From corn husks to scalable, strong, transparent bio-plastic using direct delignification-splicing strategy. *Adv. Sustainable Syst.* **6**, 2100495 (2022).

4 Arib, R. M. N., Sapuan, S. M., Ahmad, M. M. H. M., Paridah, M. T. & Zaman, H. M. D. K. Mechanical properties of pineapple leaf fibre reinforced polypropylene composites. *Mater. Design* **27**, 391-396 (2006).

5 Cheng, J. *et al.* Influences of layer thickness on the compatibility and physical properties of polycarbonate/polystyrene multilayered film via nanolayer coextrusion. *Appl. Surf. Sci.* **440**, 946-954 (2018).

6 Zheng, Y.-T., Cao, D.-R., Wang, D.-S. & Chen, J.-J. Study on the interface modification of bagasse fibre and the mechanical properties of its composite with PVC. *Compos. Part A: Appl. Sci. Manuf.* **38**, 20-25 (2007).

7 Zhang, X., Liu, W., Liu, W. & Qiu, X. High performance PVA/lignin nanocomposite films with excellent water vapor barrier and UV-shielding

properties. *Int. J. Bio. Macromol.* **142**, 551-558 (2020).

8 Kumar, M., Mohanty, S., Nayak, S. K. & Rahail Parvaiz, M. Effect of glycidyl methacrylate (GMA) on the thermal, mechanical and morphological property of biodegradable PLA/PBAT blend and its nanocomposites. *Bioresour. Technol.* **101**, 8406-8415 (2010).

9 García-Quiles, L., Fernández Cuello, Á. & Castell, P. Sustainable materials with enhanced mechanical properties based on industrial polyhydroxyalkanoates reinforced with organomodified sepiolite and montmorillonite. *Polym. Basel* **11**, 696 (2019).

10 Xia, Q. *et al.* A strong, biodegradable and recyclable lignocellulosic bioplastic. *Nat. Sustain.* **4**, 627-635 (2021).

11 Lan, K., Zhang, B. & Yao, Y. Circular utilization of urban tree waste contributes to the mitigation of climate change and eutrophication. *One Earth* **5**, 944-957 (2022).

12 Cossutta, M., McKechnie, J. & Pickering, S. J. A comparative LCA of different graphene production routes. *Green Chem.* **19**, 5874-5884 (2017).

13 Wu, F., Zhou, Z. & Hicks, A. L. Life cycle impact of titanium dioxide nanoparticle synthesis through physical, chemical, and biological routes. *Environ. Sci. Technol.* **53**, 4078-4087 (2019).

14 Van Allsburg, K. M., Tan, E. C. D., Super, J. D., Schaidle, J. A. & Baddour, F. G. Early-stage evaluation of catalyst manufacturing cost and environmental impact using CatCost. *Nat. Catal.* **5**, 342-353 (2022).
