# Supplementary material for: Turning dead leaves into an active multifunctional material as evaporator, photocatalyst, and bioplastic
Source: Nat Commun. 2023 Mar 2;14:1203. doi: 10.1038/s41467-023-36783-8 (PMC9981597; doi:10.1038/s41467-023-36783-8)
Supplement: Supplementary file 2 — Description of Additional Supplementary Files [file 41467_2023_36783_MOESM2_ESM.pdf]

Description of Additional Supplementary Files

File Name: Supplementary Data 1
Description: Atomic coordinates of lignin-cellulose-whewellite composite

File Name: Supplementary Data 2
Description: Atomic coordinates of lignin-cellulose composite
